# Intraocular lens simulator using computational holographic display for cataract patients

**DOI:** 10.1371/journal.pone.0295215

**Published:** 2024-10-23

**Authors:** Deniz Akyazi, Ugur Aygun, Afsun Sahin, Hakan Urey

**Affiliations:** 1 Department of Electrical and Electronics Engineering, Koç University, Istanbul, Turkiye; 2 School of Medicine, Koç University, Istanbul, Turkiye; 3 Research Center for Translational Medicine, Koç University, Istanbul, Turkiye; Help Me See, Instituto Mexicano de Oftalmologia and University of Miami, MEXICO

## Abstract

**Purpose:**

To develop and validate a holography based vision simulator for the demonstration of expected postoperative vision corresponding to monofocal and multifocal intraocular lenses (IOL) to cataract patients before surgery.

**Methods:**

An artificial eye model is used to measure the optical performance of different IOL types. The resultant aberrations and degradations are then modeled using phase holograms and shown to subjects on a holographic display. We measure the contrast and resolution loss, halos around the light sources, and point spread function (PSF) corresponding to three different IOLs. We tested the holography based vision simulator on 13 healthy subjects and 6 cataract patients.

**Results:**

Monofocal, bifocal, and trifocal IOLs exhibited a contrast decrease of 5%, 42%, and 45% and a resolution limit of 4.49, 4.00, and 4.00 lp/mm (using 0.05 MTF criteria), respectively. Monofocal IOLs have the best resolution and contrast at the optimal focus distance, and multifocal lenses offer extended depth-of-field but exhibit prominent halos and reduced contrast/resolution.

**Conclusion:**

We confirmed that the visual functions of IOLs could be successfully modeled using phase holograms and simulated using a holographic display without using a physical IOL. Patients can experience the effects of different IOL options prior to surgery, which helps with IOL selection, expectation management, and patient satisfaction.

## Introduction

Cataract treatment involves surgery in which a surgeon removes the opaque lens and implants an intraocular lens (IOL) in the patient under local anesthesia [1]. There are various choices for IOLs. Monofocal IOLs with a fixed focal length have been a common choice. However, there are also multifocal IOLs, engineered to provide good unaided vision for both distant and near objects. These multifocal IOLs can have two foci (bifocal), three foci (trifocal), or extended depth-of-field (EDOF). Despite their design to provide multiple foci for clear vision without glasses and their overall good optical performance, multifocal IOLs have been associated with undesirable visual phenomena. In contrast, with monofocal IOLs, undesirable visual phenomena have been observed much less frequently in previous studies [2]. The incidence of postoperative complaints related to photic phenomena and side effects continues to be one of the main concerns associated with multifocal IOLs [2, 3]. IOL technology is evolving and numerous new IOLs are coming to market, there is a dire need for a technology to characterize and simulate the vision through any IOL to be shown to patients prior to surgery.

There are a number of IOL simulators to show vision through IOLs to cataract patients prior to surgery as a solution technology [4–9]. However, they do not work well for patients with moderate or dense cataractous lenses due to impaired vision and heavy scattering. One such case is an IOL simulator that uses a biconcave lens back-to-back with an IOL placed in a thin wet chamber. The compact lens assembly has a limited field of view but allows patients to see the world through a multifocal intraocular lens [4]. Similarly, Rassow’s telescope follows the same architecture as the intraocular lens simulator but utilizes a convex lens [5]. VirtIOL (10Lens S.L.U., Barcelona, Spain) is another device for simulating vision through intraocular lenses [6]. It allows the subject to obtain the defocus curves of patients as if they were wearing the IOL of their choice. SimVis (2EYEVISION, Madrid, Spain) [7] and VAO (VOPTICA, Madrid, pain) [8] developed and commercialized cataract simulators. However, SimVis doesn’t work if a patient has dense cataracts, and VAO was tested using phase screens, but there were no clinical results for their wavefront sensor technology in dense cataracts. Also, our group developed a preoperative vision simulator for cataract patients, which involved using a small and steerable exit pupil holographic display [9], which worked well in dense cataracts but was limited to the simulation of monofocal IOLs.

In this paper, our aim was to validate our holography based vision simulator for cataract patients using measured performances of monofocal and multifocal IOLs. \ This is the first study to transform the measurement results of physical IOLs into a vision simulator using a computational holographic display and test them on cataract patients regardless of their cataract density. In this study, (i) We performed optical characterizations of different types of IOLs using an artificial eye model. Our approach enabled us to preoperatively estimate the photic phenomena and side effects that patients might experience after IOL implantations, especially multifocal IOL. (ii) We modeled the characterizations using phase holograms and incorporated them into a holographic display, building upon the preoperative vision simulator our group previously utilized for monofocal IOLs. This approach’s uniqueness is bypassing the patient’s cataract by utilizing holographic display technology, overcoming the inherent problems of other simulators, and correctly showing them postoperative vision through any choice of IOL. (iii) To validate our approach and confirm that holographic display based simulators can be used as a tool for providing a vision as if seen through IOLs, we test and compare visual acuity, contrast sensitivity, and halo severity through the holographic display simulator with healthy subjects and cataract patients. Our study suggests that our approach has the potential to provide patients with a more realistic and personalized understanding of the effects of cataract surgery and IOL implantation on their vision.

## Materials and methods

### Optical characterization setup

We used a 3D printed artificial eye model to objectively test the effects of IOL on human vision [10]. Our group adapted this artificial eye model from a previous model developed by Gobbi et al. and included a scleral lens, which is a spherical surface made of PMMA, which is compatible with the ISO 11979–2 recommendations for IOL evaluation [11, 12]. Several conventional benchtop measurements of IOL performance as well as point spread functions (PSFs) [13] associated with the chosen IOLs were assessed using the artificial eye model forming an image on a transparent glass surface, which is imaged with variable focal length camera lens (f = 12 mm to infinity, manufactured by Kowa, Düsseldorf, Germany) onto a scientific camera (3-sensor R-G-B prism area scan camera manufactured by JAI A/S, Valby, Denmark) (S1 Fig). The resulting field of view of the setup was 25 degrees, and for benchtop measurements, the pupil diameter was set at a standardized 3 mm [14]. To minimize the limitations in point spread function measurement accuracy due to the dynamic range of the scientific camera, all captures were done in 12-bit color depth mode, and the captures were then scaled down to 8-bit for the analysis.

### Optical characterization

The described setup was used to test and characterize three types of IOLs: (i) Tecnis monofocal IOL, (ii) Tecnis multifocal IOL (with +3.25D add) as bifocal IOL, and (iii) Alcon Panoptix IOL (with +2.17D and +3.25D add) as trifocal IOL, each having a base power of +15D diopter. These IOL models were selected for testing and characterization because they are widely used in cataract surgeries. We characterized IOL performance by using standard test charts such as the standard Turkish reading chart (DEVA Oftalmoloji, Istanbul, Turkiye), the USAF 1951 glass slide resolution target (Edmund Optics, Barrington, NJ), and a light-emitting diode (LED) point light source.

The corresponding point spread functions of the IOLs were found by using spherical trial lenses with powers ranging from +1.0 to -5.0 D with 1.0 D increments and the reading chart, which was placed 35 cm away from the setup. The focal length of the camera lens was set such that the camera was focused on the reading chart with the 0D lens. The resulting images of the reading chart with increments of 1D were captured for each IOL. The point spread functions of each IOL for diopter powers from +1.0 to -5.0 D were calculated by a MATLAB script, taking the captured reading chart with a standard lens as the reference image.

The resolution efficiency [15] and contrast decrease characterizations were done with the USAF 1951 target captures through the artificial eye model setup. The USAF 1951 target was placed 35 cm away from the setup, and for each IOL, the focal length of the camera lens was varied until the best corrected focus was found. Then, captures were evaluated for the least achievable contrast limit to resolve two separate lines by human eyes, which was chosen as a 0.05 MTF limit [14]. The magnification changes of the images induced by changing the focal length of the camera lens was also considered when the evaluation was done.

Contrast decrease characterization was calculated using the same captures as the USAF 1951 target and obtaining MTF graphs of the IOLs. To eliminate the variations in contrast ratio, it was calculated in each resolution group of the chart for separate orientations at 18 intersections, and then the results were averaged. These calculations were performed starting from Group 0, Element 1 (1.00 lp/mm), to Group 2, Element 3 (5.04 lp/mm), and obtained a contrast ratio for each element of the chart using the Michelson contrast formula [15]:

$$C=\frac{L_{max}-L_{min}}{L_{max}+L_{min}}$$ (1)

where L_max_ is the maximum intensity on the white side of the intersection and L_min_ is the minimum intensity on the black side of the intersection.

The USAF 1951 target was also captured without an IOL in the setup to eliminate any factors other than the IOL that could impact the decrease in contrast, such as external factors and camera quality. The same contrast calculation was conducted on this capture, and the best contrast achieved from this capture was rounded to a 1.0 contrast ratio. Then, contrast calculation results from IOL captures were rounded accordingly. Taking the magnification change due to the camera lens and the distance of the USAF 1951 target from the setup into account, spatial resolution was calculated in terms of cycles per degree (cpd) to plot the contrast decrease curves.

An LED light source (KY-009 RGB) was placed 1 meter away from the artificial eye model setup to objectively evaluate the occurrence and characteristics of photic phenomena created by IOLs. Three captures were taken without adjusting the camera settings for each IOL, with LED colors of red, green, and blue. Saturated results were avoided by employing neutral-density filters in the setup to ensure objectivity. The size of the photic phenomenon in captured images was found by converting the pixel sizes to their corresponding physical sizes. Assuming that the photic phenomena were nearly perfect circles, the radial average of their intensities was calculated and plotted at their physical locations, clearly showing the intensities of the peak and halos as well as the size of the glare.

### Holographic display simulator

Holographic displays offer unique capabilities for direct view displays and virtual and augmented reality applications. Among all technologies, holography stands out as the sole technology capable of generating programmable exit pupil patterns [16]. In a prior study, our group leveraged this characteristic to conduct preoperative vision screening combined with cataract screening and verification for cataract patients. This screening involved the implementation of a distinct approach known as the eye-box steering method, previously developed by our group [9].

In this study, the aim was to expand upon the previous experiments conducted by our group. The characterized effects of IOLs are introduced to the visual assessment, thereby achieving postoperative vision while considering the additional side effects introduced by the IOLs to the eye.

#### Applying optical characterizations to target contents

After characterizing and measuring the point spread function of the selected IOLs, the subsequent step of the study involved applying them to suitable images and conventional testing charts commonly used in ophthalmology examinations. Three different approaches were employed to accurately recreate the desired image quality.

For the visual acuity assessment of the subjects, the point spread functions obtained from the captures of the reading test were convolved with a pre-prepared Snellen chart. This convolution process generated 18 chart images, corresponding to different vergences ranging from +1.0 to -5.0 for all three IOLs, while creating an appropriate decrease in resolution. These charts were utilized during the evaluation of visual acuity.

To specifically evaluate the decrease in contrast sensitivity induced by the IOLs, the contrast of target sinewave grating patterns was manipulated based on the characterization performed using the artificial eye model. A total of 20 sinewave grating targets with spatial frequencies of 3, 6, 9, 12, and 18 cycles per degree (cpd) were adjusted accordingly, corresponding to the contrast reduction caused by the IOL at each specific frequency. Each spatial frequency content consisted of five samples of contrast thresholds, ranging from higher to lower contrasts calculated using Michelson’s formula [17].

**Fig 1:**
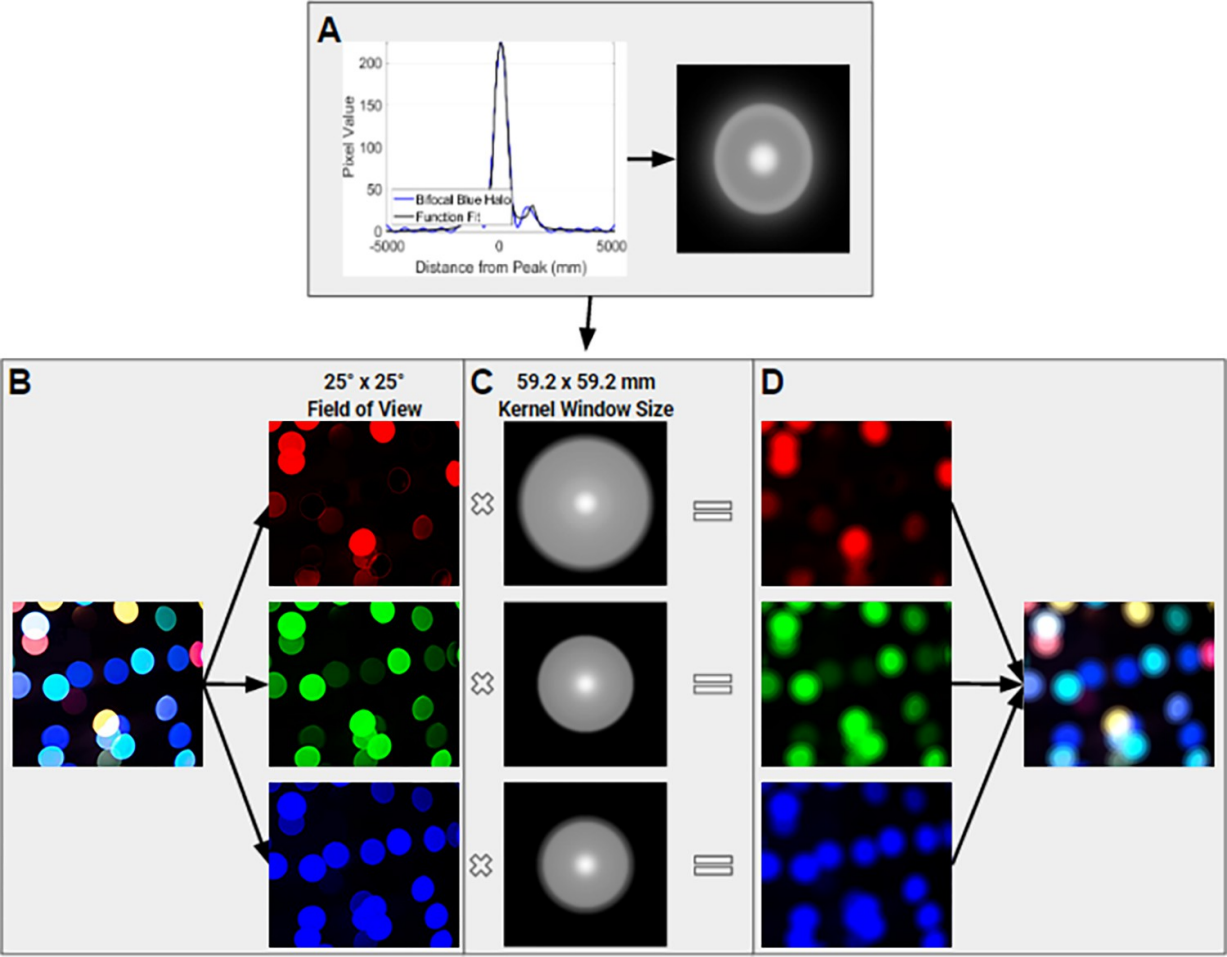
Flowchart of steps for applying IOL effects to a target image. (A) A function fit and radial averaging are applied to pixel intensity plots, and the cross-section is converted into a radially symmetric 2D simulated PSF of the corresponding halo pattern; (B) the target scene is separated into 3 color channels (red, green, and blue); (C) the simulated PSF of the IOL for each color; and (D) the results of each channel are combined to achieve a full color scene.

In order to simulate precise photic phenomena through convolution, the approach used for the Snellen chart was extended. Initially, a function fit was applied to the photic phenomena observed in the captures of LEDs to express the effects of the IOLs on a point light source, resulting in a radially symmetric two-dimensional point spread function for each color. The target image was then separated into its three-color channels (red, green, and blue), and each channel was convolved with the corresponding point spread function. Finally, the results of each convolution were combined to create a full-color scene with the desired photic phenomena (refer to Fig 1).

#### Computer-generated holography calculation

To utilize the holographic display simulator, recreated images are calculated to corresponding phase images through computer-generated holography (CGH) calculation with an algorithm developed by our group previously [18]. Through this calculation, complete computer control over the holographic display is achieved. The CGH calculation used in this study consists of several steps. After the recreated image and its phase map are given to the algorithm as input, the image is discretized into various focus planes based on depth map values through iterative Fourier transform algorithms [18], and the object wave on the scene plane is computed using Fresnel Space Propagation, considering the optical parameters of the display setup [19]. In the concluding step, the complex-valued object wave is determined. The target content is replicated through the generation of encoded CGH. A holographic display setup forming a steerable small exit pupil was utilized to present the encoded CGH to the subject through a 1 mm exit pupil, which was then steered on the subject’s pupil until a non-cataractous area was found based on the eye-box steering method [9].

Although utilizing the pinhole display overcomes the cataract in the subjects, a small error may still occur due to refractive error on the subject’s crystalline lens. To minimize this error and get more accurate results, a refractive correction algorithm was used on the calculated holographic contents, which was extensively discussed by our group previously [9].

#### Holographic display architecture

**Fig 2:**
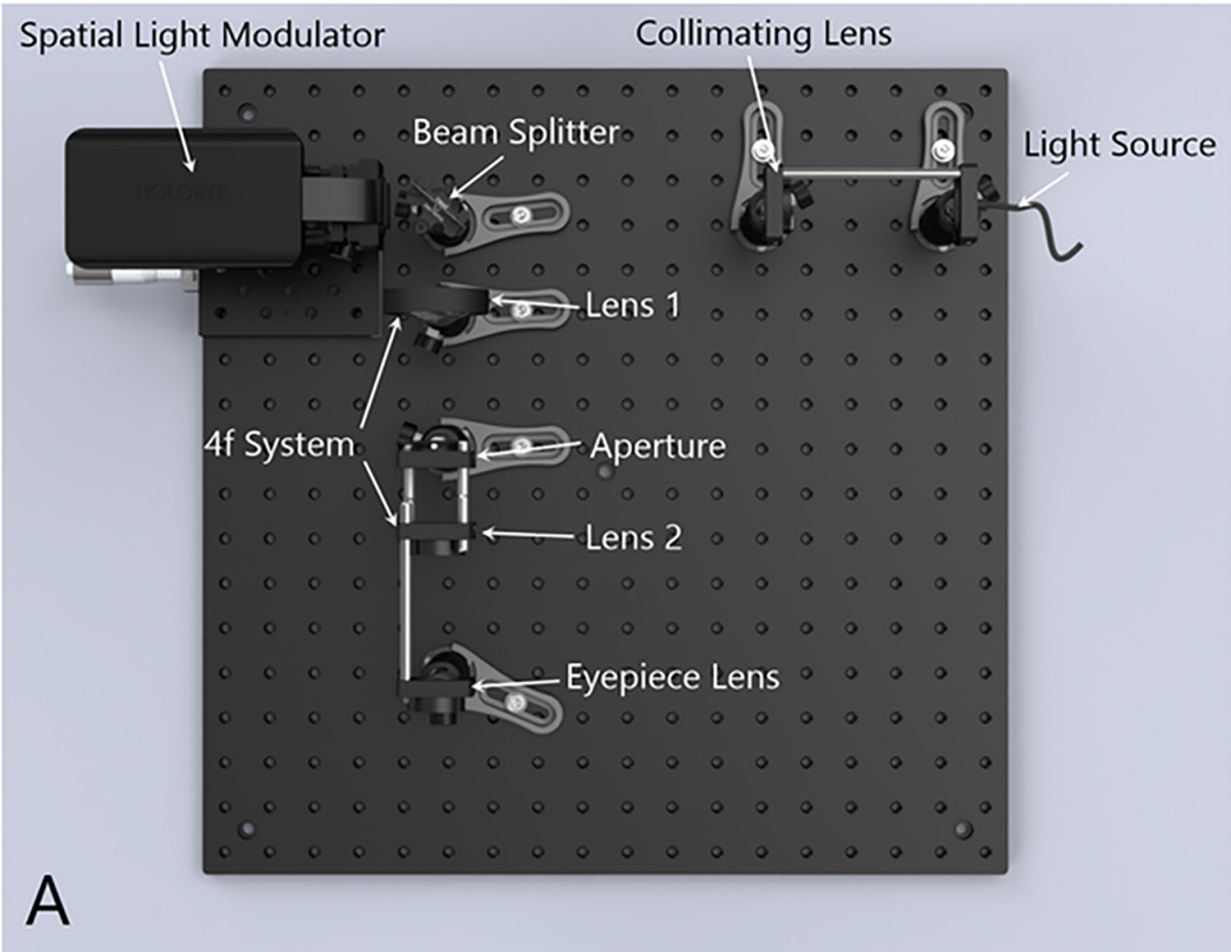
Holographic display setup. All assessments were conducted on a proof-of-concept holographic display setup.

The holographic display setup consists of a point light source, a custom phase only SLM, and optical components (Fig 2). A 35 mm focus convex lens (Thorlabs, Newton, NJ) collimates the light coming from the point light source, which then illuminates the SLM. The operating light wavelengths are 470 nm for blue, 530 nm for green, and 632 nm for red. The SLM modulates the light with a resolution of full high definition (1920 x 1080 pixels) and a pixel pitch of 4.5 micrometers. The propagated light beam then goes through a 4f-lens system consisting of two lenses (Thorlabs, Newton, NJ) of 50 mm focal length, respectively, and an aperture that acts like a spatial filter. Finally, the beam goes through another lens with a focal length of 50 mm to reach the target magnification and reaches the patient’s eye. The holographic display’s field of view is 6 degrees, according to the optical components’ parameters.

### Patient tests

The study was conducted following the institutional guidelines and received approval from the Koç University Ethics Committee (Approval number: 2019.400.IRB2.121) for human subject research in accordance with the principles of the Declaration of Helsinki. Prior to the testing, the subjects were informed about the purpose and methodology of the study, and their informed written consent was obtained. A diverse sample of 17 subjects (ages 23–65) was recruited between May 2023 and September 2023 for clinical validation studies. The medical records used in this study were accessed on September 2, 2023, and patient information was kept anonymous.

In the test setup, the holographic display simulator was utilized to assess the subjects’ vision through IOLs. The evaluation involved measuring visual acuity, contrast sensitivity, and satisfaction with vision with photic phenomena. Holographic contents were generated to replicate the image quality experienced with the tested IOLs using techniques such as convolution or contrast manipulation discussed above. Cataract patients were tested with content computed for their IOL choice, while healthy subjects were tested with all three IOLs to enable better comparisons. To minimize bias, the test contents were randomized for a double-blinded experiment.

For the assessment of visual acuity, subjects were presented with a logMAR visual acuity chart displayed as holographic content. The chart was correctly sized for a viewing distance of 4 meters from the subjects’ eyes. Different defocus levels were simulated by incorporating the point spread functions (PSFs) corresponding to the tested IOLs. This simulated defocus was equivalent to placing a spherical trial lens with powers ranging from +1.0 to -5.0 D in front of the subjects’ eyes, in increments of 1.0 D.

To evaluate the subjects’ contrast sensitivity with IOL effects, a sinewave grating contrast sensitivity test was conducted. Holographic contents displaying sine-wave gratings with spatial frequencies of 3, 6, 9, 12, and 18 cycles per degree (cpd) were presented to the subjects at 3 meters. The contrast ratios of the gratings were adjusted according to the tested IOL’s contrast ratio corresponding to each spatial frequency, as characterized by the artificial eye model.

The evaluation of satisfaction of vision with photic phenomena was approached with some subjectivity. The subjects were presented with holograms of PSF-applied content for each IOL, including night scenes with lights and daytime scenes. The importance of exposing patients to these specific scenes was recognized, considering that most of the dissatisfaction related to halos and glares occurred at night due to lights. Subsequently, the subjects were asked to rate their satisfaction with the vision provided by the holographic contents on a scale of 1 to 5. On this scale, a rating of one indicated a state of being very dissatisfied, while a rating of five denoted a state of being very satisfied.

An extra assessment step was added for patients with cataracts, fully utilizing the holographic display’s programmable exit pupil pattern feature. A holographic content corresponding to the best visual acuity of the patient with correct spherical aberration correction was sent to the patient’s pupil and steered on a 5x5 grid with a total size of 5.25 x 5.25 mm until the region with the best visual acuity was found. The region was also tracked with densitometer data taken in real-time during the assessment, confirming that the selected pupil location corresponded to regions with the least number of cataracts. The densitometer data was compared with Scheimpflug data of the patient’s pupil to validate the results further.

### Statistical analysis

The Kolmogorov-Smirnov test was utilized to statistically analyze patient information (age, spherical equivalent, cylindrical error) and test results (contrast sensitivity, vision satisfaction) and find if they were normally distributed or not. In the case of a normal distribution, the mean plus or minus the standard deviation was employed, and the data was subjected to paired comparisons using the paired t-test. For non-normally distributed data, the median (interquartile range) was used, and paired comparisons were conducted with the Wilcoxon signed rank test. The statistical analysis was performed using MATLAB R2022a, with a significance level of 5% considered.

## Results

### Optical performance and simulation through holography

**Fig 3:**
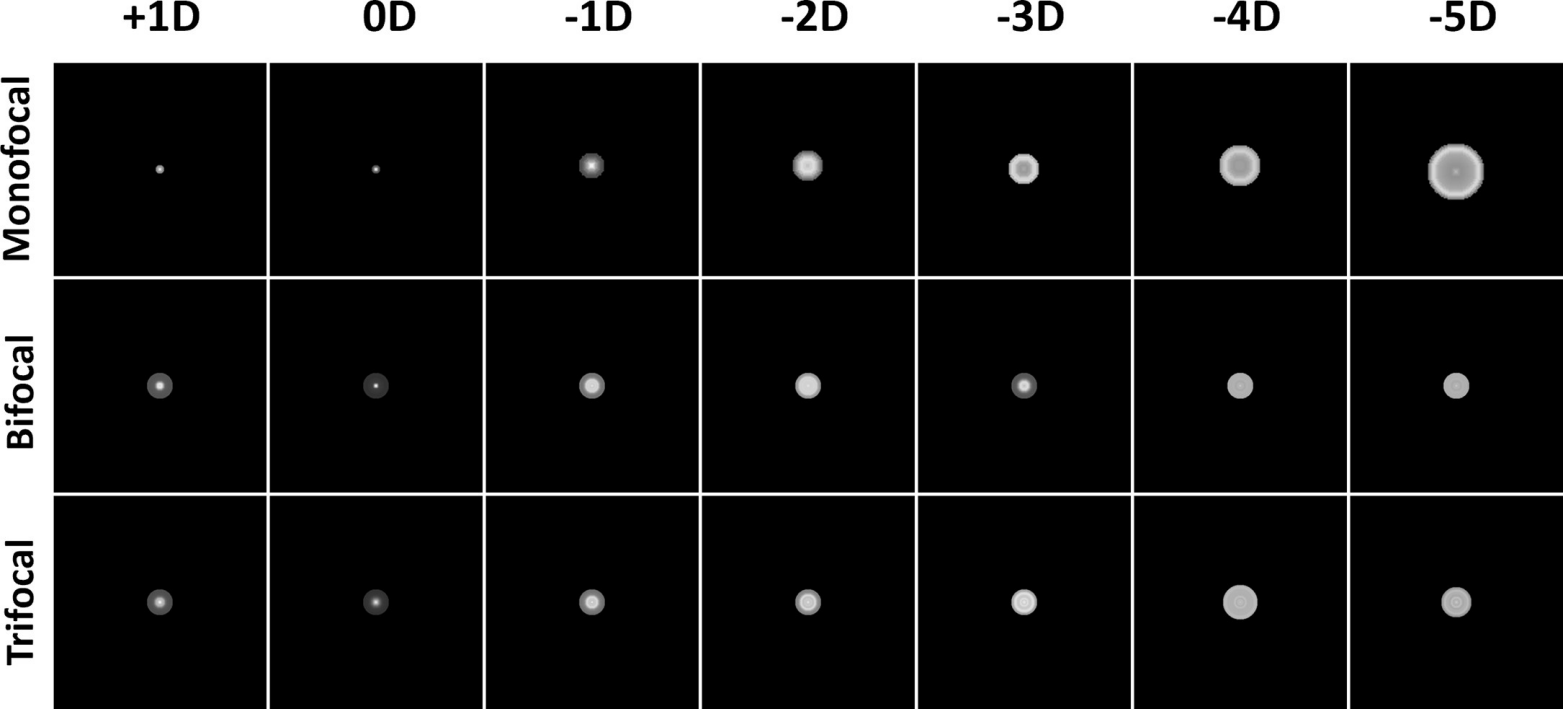
Point spread functions of intraocular lenses at different vergences. For each IOL, reading charts were captured using the artificial eye model. Resulting PSFs point out that monofocal IOL has a constantly decreasing focus starting from 0D towards -5D. Bifocal and trifocal IOLs regain their focus in -3D and -2D respectively.

The PSFs found from the captures of the reading chart are given below (Fig 3). A continuous expansion from 0D to -5D in the PSF size was observed for the monofocal IOL. A slight glare was evident in the PSF of the bifocal IOL at 0D defocus, which increased in intensity as the defocus level rose, resulting in a single halo in the PSF. Additionally, at a defocus of 3D, the light became focused at the center, leading to the emergence of a second focus within the defocus curve. Conversely, the trifocal IOL PSF exhibited a slight glare at 0D defocus but displayed the appearance of two halos in sequential order as the defocus level changed. At -2D and -3D defocus, the trifocal IOL PSF featured a single and double halo, respectively, along with a peak brightness in the center, indicating the presence of a focus at these defocus levels.

**Fig 4:**
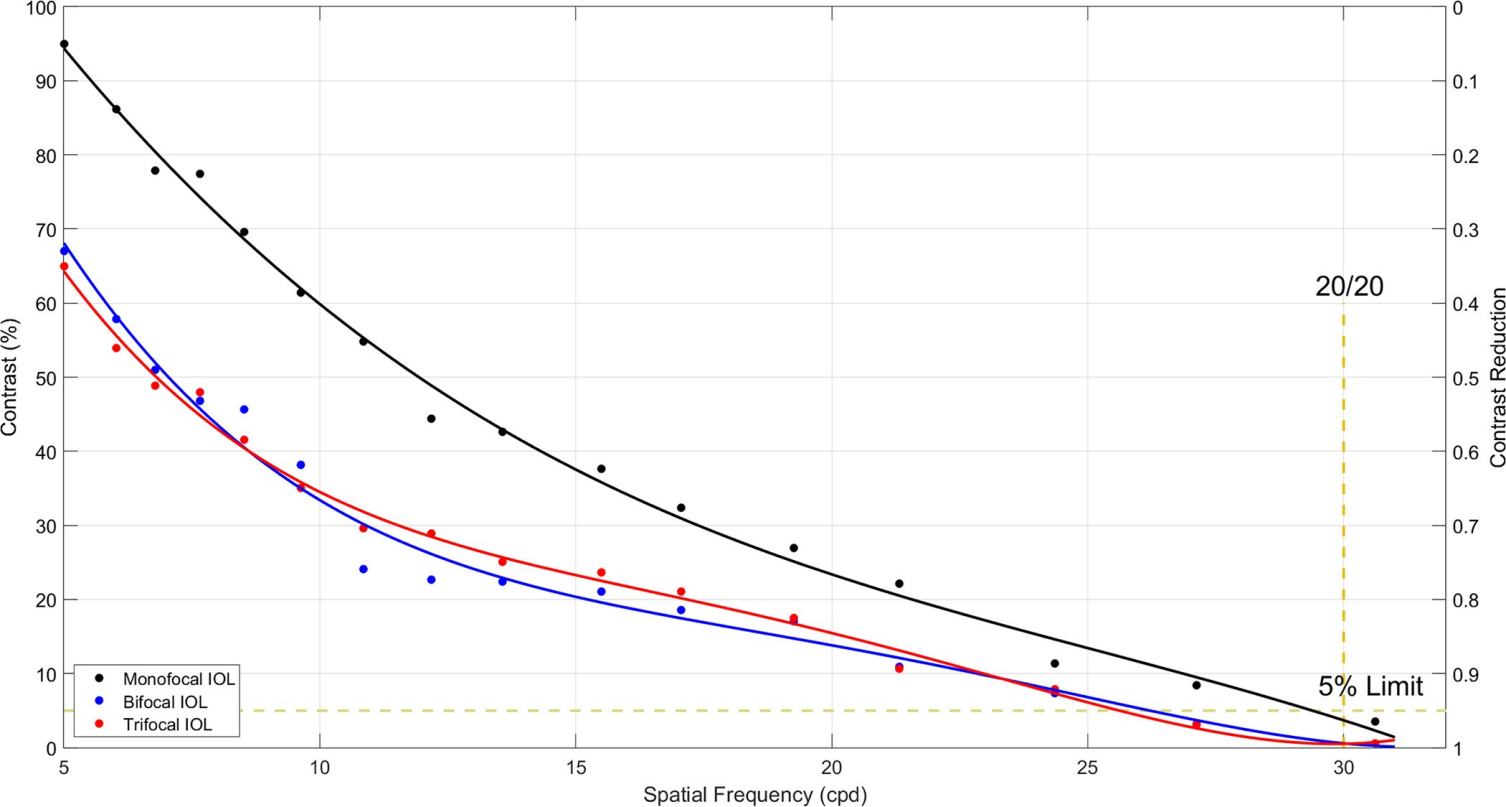
Contrast percentage curves for different IOLs as a function of spatial frequency. Bifocal and trifocal IOLs show a similar curve and less contrast compared to monofocal IOL. Using a 5% contrast limit for human eyes, monofocal IOL reaches 29.3 cpd, while bifocal and trifocal IOLs reach 26.2 and 25.6 cpd in spatial frequency.

The contrast decrease for each IOL was calculated using the USAF 1951 target (S2 Fig) as a function of spatial frequency (Fig 4). The monofocal IOL exhibited its highest contrast (95%) at approximately 5 cycles per degree (cpd) spatial frequency, with the contrast gradually decreasing as the spatial frequency increased. At around 32 cpd spatial frequency, the contrast approached nearly zero. The contrast pattern for the bifocal and trifocal IOLs followed a similar trend to that of the monofocal IOL, but with lower starting contrasts at 5 cpd spatial frequency. The bifocal IOL started with a slightly higher contrast (68%) compared to the trifocal IOL (65%). As the spatial frequency increased, both IOLs demonstrated a decreasing trend, occasionally crossing paths. The contrast lines intersected the 0.05 MTF criteria limit at 29.3, 26.2, and 25.6 cpd for monofocal, bifocal, and trifocal IOLs, respectively.

**Fig 5:**
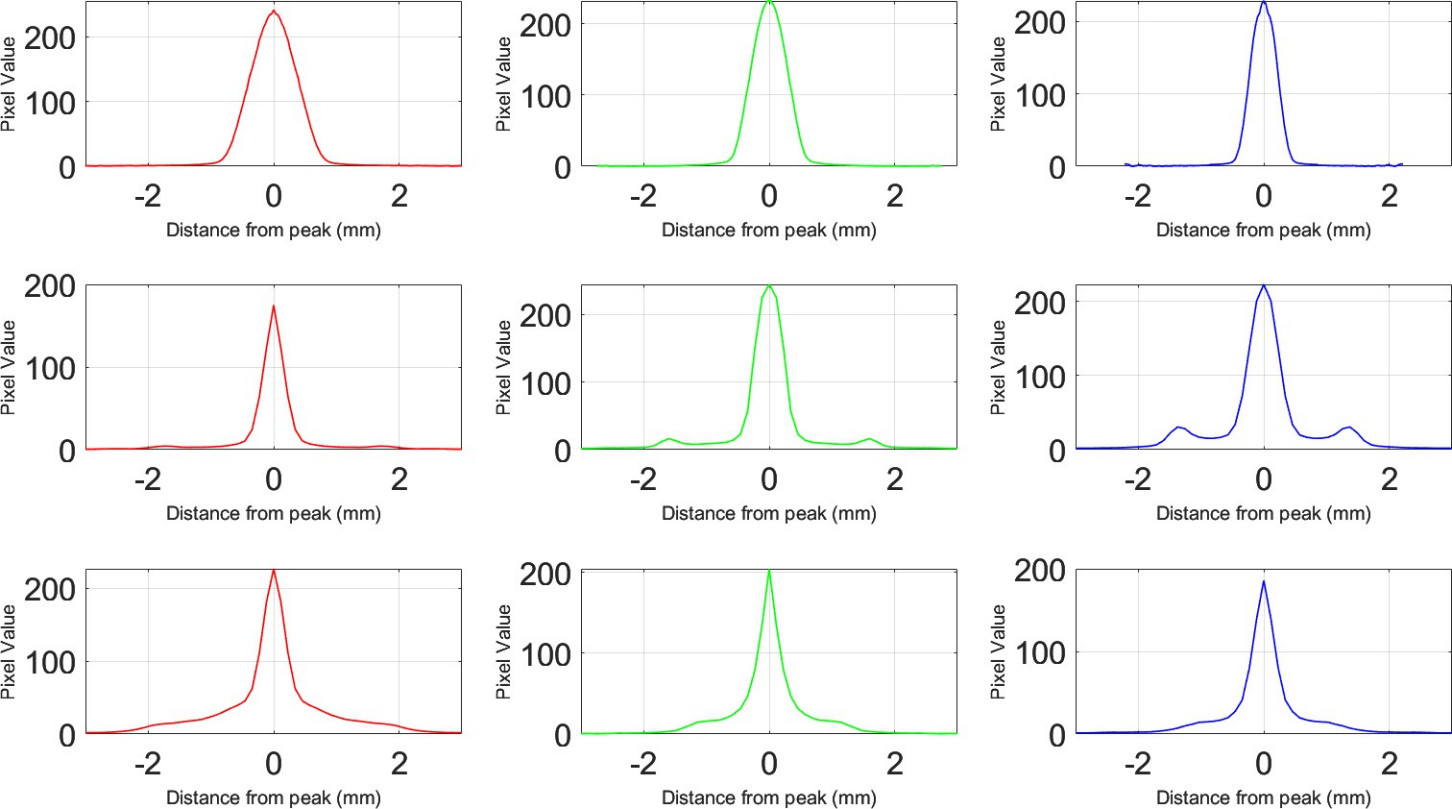
Pixel intensity plots from captures of LED light source. Monofocal IOL shows an expected peak at the center with no glare or halos. Bifocal IOL has prominent halos as small peaks around the main peak with little glare. Trifocal IOL tends to have more glare than a prominent halo. The red-light source in each case shows a wider peak compared to other colors (blue and green).

Pixel intensity plots found from capturing point light sources (S3 Fig) with different colors for each IOL are shown in Fig 5. In the plots of the monofocal IOL, visible center peaks are observed, following a normal distribution pattern. These peaks gradually decrease in size as the color transitions from red to blue. For the plots of the bifocal IOL, distinct halo peaks and glare are evident, separate from the center peak. The magnitude of the halo increases as the color shifts from red to blue, while the size of the glare decreases. Specifically, the red-light source generates a wider halo with a lower magnitude, whereas the blue-light source produces a narrower halo with a greater magnitude. The green light source falls in between these two extremes. In the plots of the trifocal IOL, glare is more prominent, while the magnitude of halos is comparatively smaller. Once again, the red-light source exhibits a broader photic phenomenon and a lower halo magnitude compared to the other colors.

As discussed in the methods section, the optical characterization results found from the artificial eye model setup were then applied to their corresponding test charts by either convolution or manipulation, prepared to be calculated as CGH content for the holographic display simulator. The resulting images from these applications are given in S1 File.

**Fig 6:**
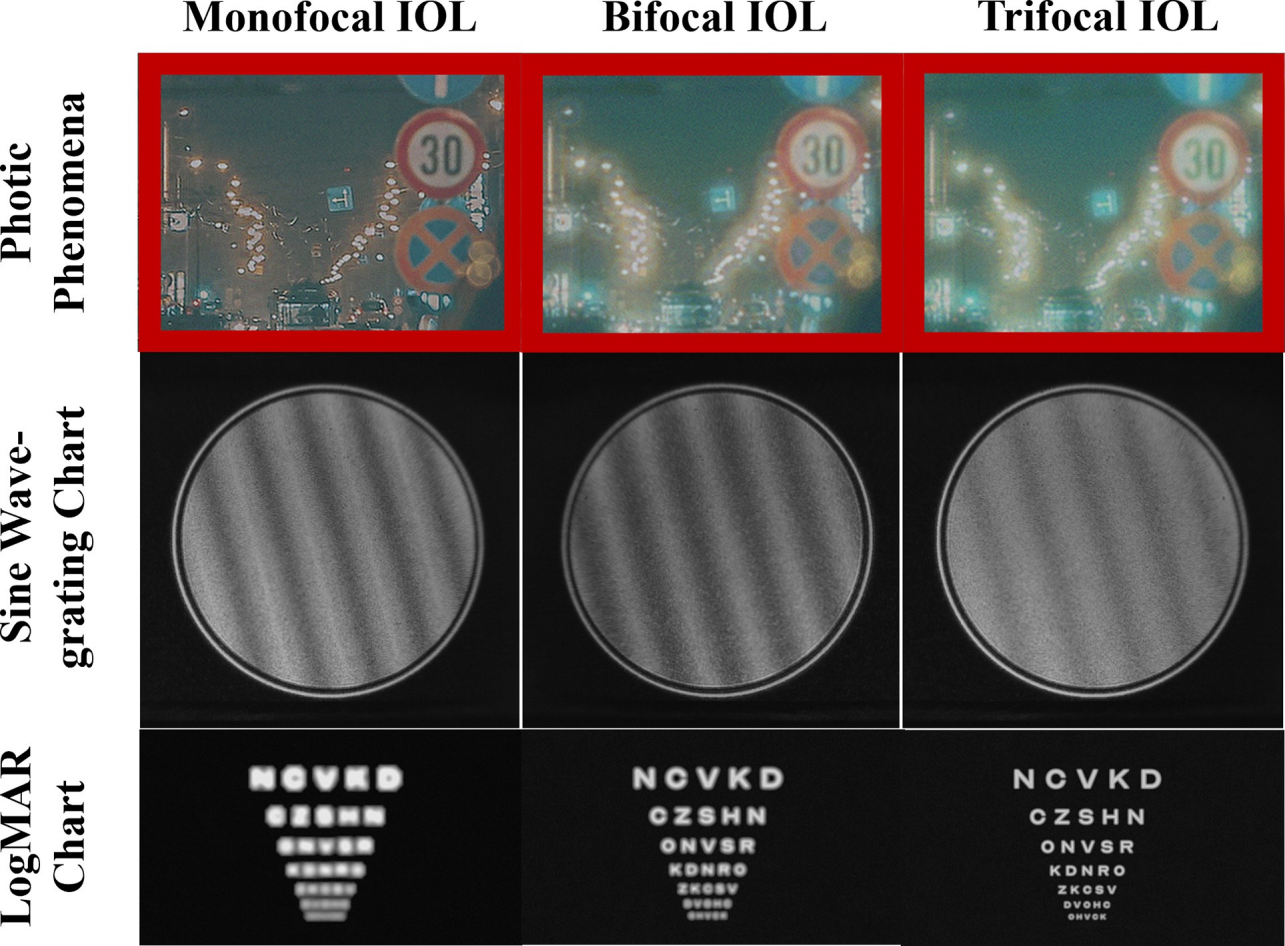
Holographic content examples for different IOLs used in the assessments. (A) LogMAR chart simulations; (B) sine wave-grating chart simulations; and (C) night-time traffic scene simulations for different IOLs (50 degrees field of view).

Some examples of calculated holographic contents as simulations by the CGH calculation to be used at the assessments are given in Fig 6. The logMAR chart holograms were calculated with the recreated chart images by convolution with the reading chart PSFs. As can be observed, charts corresponding to different IOLs appear to be resolved at different magnitudes at the same defocus level. The sine wave-grating chart holograms were calculated from the images with manipulated contrasts according to the contrast decrease curves. Again, although the target image was the same, due to contrast manipulation, different IOLs appear to have different contrast ratios. The example scene holograms with photic phenomena were calculated from the scenes created by convolving original images with corresponding PSFs obtained from the LED light source. Some contrast decrease is observed in the results; when compared to the monofocal IOL, in addition to blur and contrast decrease, bifocal and trifocal IOL PSFs add extra halos and glare, becoming a visible discomfort for the patients. It is important to note that since the main aim of these scenes was the photic phenomena and side effects, the applied PSFs were found from the best focuses reached with each IOL, and the focusing of the images is limited in showing the actual results.

### Validation tests for holographic display simulator

Thirteen eyes from thirteen healthy subjects and six eyes from six patients with various degrees of cataracts were included in this study. Relevant details of the subjects are given in Table 1. The median age of the subjects was 35.0, and the gender distribution was 15 men to 4 women. In the assessments done on subjects with cataracts, the non-cataractous regions found with the eye-box steering method were validated through comparison to the Scheimpflug Cataract Densitometer results of the subject.

**Table 1:** Demographics and characteristics of the subjects participating in the study.

	Parameters
**Age, years (mean, range)**	42, 50
**Gender (men: women)**	15:4
**Spherical equivalent, D (mean ± SD)**	-1.4 ± 2.8
**Cylindrical, D (median (IQR))**	0.5 (0.75)

D = diopters, SD = standard deviation, IQR = interquartile range

**Fig 7:**
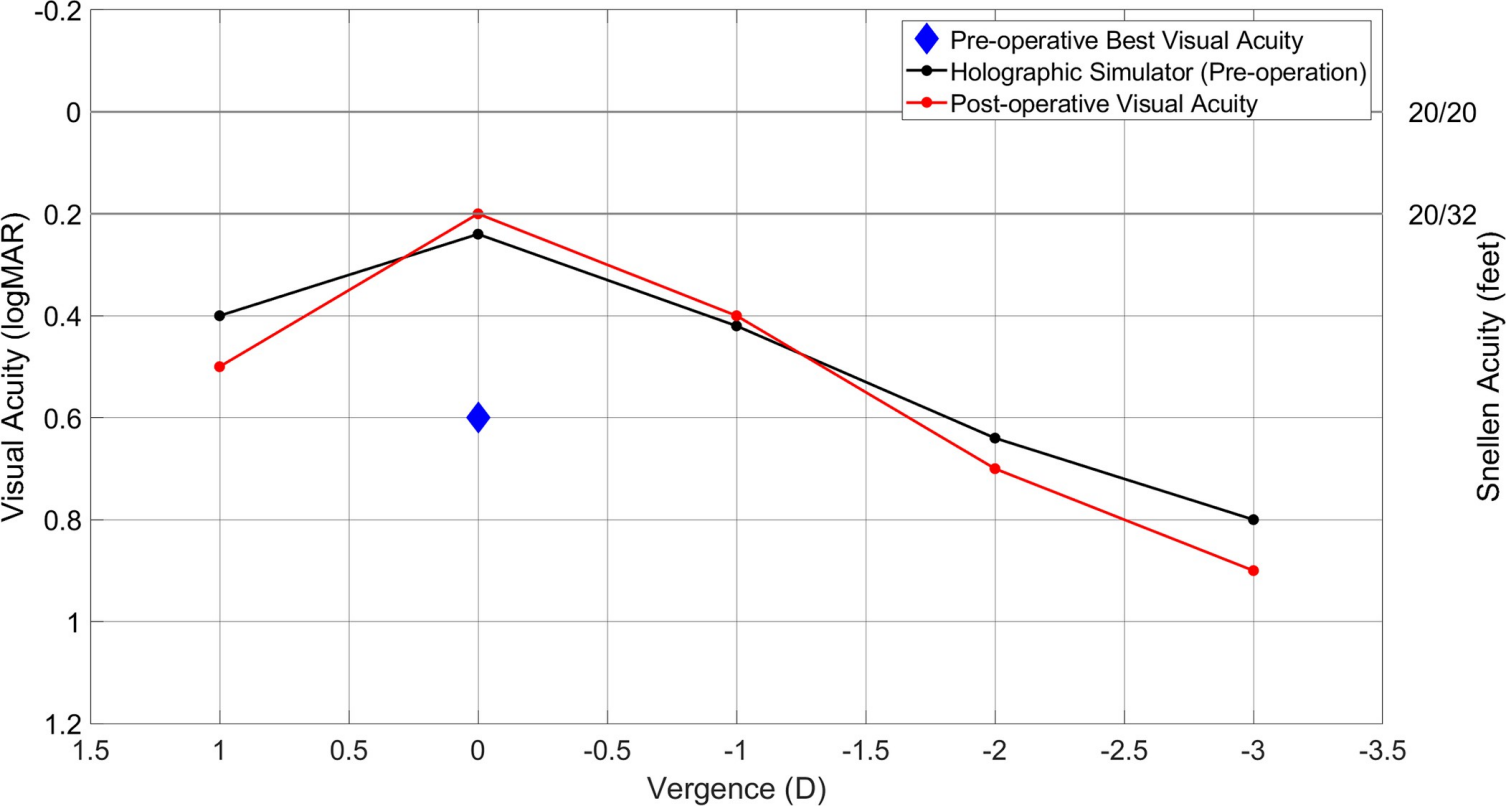
Visual acuity results of holographic (pre-operation) and clinical (post-operation) assessments done with the patient 1 month after monofocal IOL implantation.

**Fig 8:**
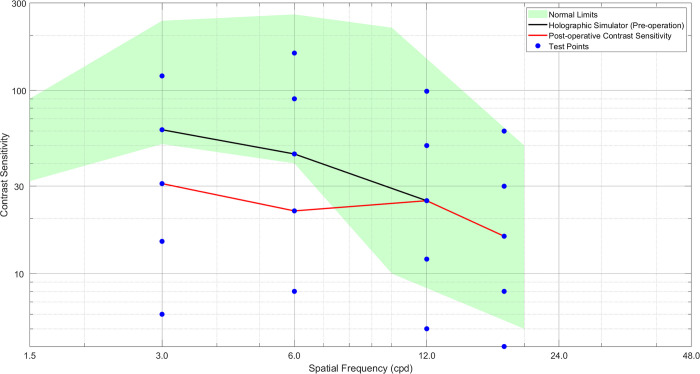
Sinewave contrast sensitivity results of holographic (pre-operation) and clinical (post-operation) assessments done with the patient 1 month after monofocal IOL implantation.

The defocus curve and contrast sensitivity results obtained from the holographic simulator and clinical evaluations conducted on a cataract patient are depicted in Figs 7 and 8. The holographic simulator measurements were carried out before the surgical procedure, while the clinical assessments were performed four weeks after the patient received a monofocal IOL implant.

In Fig 7, the defocus curves for both sets of assessments are presented alongside the best visual acuity measured using conventional methods before the surgery. Notably, the best visual acuity achieved with the holographic simulator and the post-operative clinical measurements is approximately 0.2 logMAR at 0D. In contrast, conventional methods fall significantly short of achieving comparable visual acuity due to their inability to address the patient’s cataracts, showing 0.6 logMAR measurements. In both graphical representations, the peak visual acuity occurs at 0D, and as the vergence decreases, both datasets exhibit a decline in visual acuity following a similar pattern.

Fig 8 illustrates the outcomes of contrast sensitivity assessments using sinewave grating tests conducted with the holographic display simulator and clinical evaluation 1 month after surgery. Notably, at lower spatial frequencies, the holographic simulator results indicate one test point higher contrast sensitivity compared to the post-operative clinical assessment findings. However, both sets of measurements converge at higher spatial frequencies, showing similar contrast sensitivity values. The observed disparity between the holographic and clinical assessments may be attributed to the patient’s ongoing adaptation to their new visual circumstances. Given that the adaptation period can vary among individual patients, it is plausible that the tested patient had yet to attain their optimal stable vision during the evaluation. Consequently, in subsequent assessments, the patient may eventually achieve contrast sensitivity levels akin to those obtained through the holographic simulator in their clinical assessments.

Detailed results of assessments done on healthy subjects and cataract patients for various IOLs, and their statistical analysis are given in the S2 File.

Table 2 shows the halo distance from the center and the halo magnitude relative to the center peak, which were calculated from the artificial eye model characterizations. The last row shows ratings of preoperative vision satisfaction provided by cataract patients after experiencing simulated vision with corresponding IOLs, assessed on a scale from 1 to 5. This scale was selected because it is commonly used in ophthalmology clinics and it makes patient feedback easy to obtain. The bifocal IOL had a greater halo distance from the center than the trifocal IOL, while the trifocal IOL had a greater halo magnitude relative to the center peak. For satisfaction with vision under photic phenomena, the monofocal IOL again was better than the other IOLs (P < 0.01, paired t-test, for both cases). Neither the bifocal IOL nor the trifocal IOL satisfaction was statistically better than the other (P = 0.096, paired t-test).

**Table 2:** Characteristics of photic phenomena and satisfaction survey results.

	Monofocal IOL	Bifocal IOL	Trifocal IOL	p-value		
				Mono and Bi	Mono and Tri	Bi and Tri
Halo distance from center, mm (cpd)	-	1.60 (313.5)	1.33 (382.9)	-	-	-
Halo magnitude relative to center peak (%)	-	7.6	9.4	-	-	-
Satisfaction with simulator vision, 1–5	4.15 ± 0.38^a^	2.00 ± 1.22^a^	2.38 ± 1.19^a^	<0.01^b^	<0.01^b^	0.096^b^

Mm = millimeters; cpd = cycles per degree; IQR = interquartile range; Mono = monofocal; Bi = bifocal; Tri = trifocal.

^a^Mean ± standard deviation

^b^Paired t-test

## Discussion

To our knowledge, we are the first ones to apply the characterizations made by an artificial eye model to holographic content and show the results to subjects. Experiencing the vision of an IOL through a holographic simulator helps subjects understand the effects of their IOL choice and shows them what to expect after surgery.

Existing simulators in the literature lack the ability to overcome the cataracts of subjects, especially in dense cataract scenarios. Our approach enables us to create a holographically formed image on the retina through cataractous lens of the patients. Furthermore the holographic images are computed using the measured performance of monofocal as well as multifocal IOLs. However, some limitations still exist with this approach.

The use of scientific cameras had negative effects on finding the actual PSF of the IOLs in a human eye. A camera has a very limited dynamic range compared to the human eye and is not able to capture all the details. In this study, the captures for characterization were taken in 12-bit raw images to minimize this limitation. This was enough for the purpose of estimating the visual results, but for a more accurate characterization, a wavefront sensor instead of a camera could be used with the same methods [13]. Regardless, the PSF functions obtained were similar to those obtained in previous studies [13, 20].

An additional noteworthy limitation encountered in our study pertained to the saturation levels observed in the initial image captures, which were subjected to PSF convolutions. Despite the digital single-lens reflex camera employed possessing a dynamic range of 16 bits, the acquisition of completely unsaturated nighttime scenes was unattainable due to the persisting scientific research problem regarding the capture of unsaturated nighttime images with lights [21] and 16 bits being still very limited compared to the dynamic range of the human eye [22]. This inherent limitation led to preexisting glares present in the original captures prior to convolution, thereby inadequately capturing the comprehensive photic phenomena induced by IOLs in human vision.

The scleral lens is a spherical surface and is included as part of our model in this study. The profile differences between the subjects’ cornea and the scleral lens can be incorporated into the model once they are measured for the subject. Such differences can be neglected in most cases since our evaluation is done using a holographic display system with a small exit pupil size (1–2 mm). Furthermore, when there are significant corneal aberrations (i.e., astigmatism and higher order aberrations), clinicians can account for them in the IOL selection (e.g., toric IOLs). The PSFs used in our model can be adjusted based on corneal aberrations if they are substantial. None of the subjects evaluated in our case had higher-order corneal aberrations, so it was not necessary in the current study.

In the contrast decrease characterization and simulation, rather than the PSF, the contrast decrease curves were used. This approach didn’t create a significant difference in the contrast sensitivity results (a maximum of one test point difference). We believe applying the obtained PSFs to corresponding sine wave-grating charts would lead to similar results, but this was not investigated in this study.

The human eye, IOLs and eye models all suffer from longitudinal chromatic aberration (LCA). LCA is more complicated for multifocal (refractive-diffractive) IOLs and is typically optimized for approximately 555nm [23]. We tested defocus performance and contrast sensitivity using a green LED centered at 530nm, and as such, chromatic aberrations did not impact our study. Our study and methodology can be extended to other wavelengths and polychromatic illuminations.

Our CGH computation algorithms are optimized to eliminate coherent artifacts (e.g., laser speckle) by using engineered phase factors rather than random phases, and our simulator uses small LED light sources instead of Lasers [24, 25].

Although holographic displays have the ability to create virtual images with all the 3-dimensional (3D) depth cues, this ability was not utilized in this study since the common ophthalmologic examinations are done with 2-dimensional contents. This study focused on displaying the content at different depths, so it matched ophthalmologic examinations better while bypassing cataracts. In photic phenomena simulations, it was treated as if the sources of the phenomena were in a single distant plane from the subject’s eyes, convolving the original image with a PSF corresponding to a single chosen depth plane. While this was sufficient to show the IOL effects over point light sources and give the subjects an idea, it didn’t fully simulate how light sources at different depths would be seen after the operation.

If vision through an IOL wanted to be fully simulated, a different approach could be taken. In the characterizations, scenes with different depths could be captured by the artificial eye model, and PSF for the respective depths could be obtained. Then, a scene image with the same depths could be used, with corresponding parts of the image for different depth PSFs separately convolved and combined to reach 3D simulation results.

In the assessments of IOL vision through our simulator, fully corrected visual acuity, contrast sensitivity, and satisfaction with the vision of subjects were tested with the vision simulator. The defocus curves measured using our holographic display gave similar results to the defocus curves of patients obtained clinically after cataract surgery with corresponding IOLs [26, 27]. The contrast sensitivity results we obtained using the vision simulator well match the clinical postoperative contrast studies of the IOLs in patients [27, 28]. Regardless, the results of the test showed that photic phenomena decreased the visual acuity of the subjects. After seeing the photic phenomena and side effects of IOLs on our simulator, subjects were more satisfied with monofocal IOLs compared to multifocal IOLs (bifocal and trifocal IOLs). Our survey results also agreed with previous IOL simulator studies with different methods [4].

## Conclusions

This study has 3 main contributions, (i) we executed comprehensive optical characterizations of physical IOLs, (ii) transposed these characterizations into phase holograms for vision simulation, and (iii) validated this methodology with cataract patients, thereby affirming holography as a viable solution for IOL simulation. In conclusion, this is the first study to transform the measurement results of physical IOLs into a vision simulator using a computational holographic display. Our vision simulator allows cataract patients to experience postoperative vision and photic phenomena associated with different IOL options. The test results for defocus curve, contrast sensitivity, and satisfaction matched the results of the aforementioned studies in the literature and were further confirmed with the clinical results in our limited studies with cataract patients. Using the combined methods of an artificial eye model and holographic display, regardless of the severity of their cataracts, patients could experience the side effects as well as the function of any IOLs before their surgery and know what to expect from their choice of IOL. This vision simulator is anticipated to serve as an important research tool for IOL manufacturers and vision scientists, an educational tool for ophthalmologists, and a decision support system for patients in cataract surgery clinics.

## Supporting information

S1 Fig Artificial eye model setup. (A) A real-time capture of the scientific camera combined with an artificial eye model is given. We kept the system as compact as possible to avoid ambient light and optical aberrations from the environment. (B) An example setup used for IOL characterization. To correct the magnification, we used an artificial eye model and a camera with a x10 microscope lens.
(TIF)

S2 Fig USAF resolution charts photographed with artificial eye model setup, and corresponding pixel intensities through an example cross-section. Monofocal IOL (A) had better focus compared to bifocal IOL (B) and Trifocal IOL (C). Pixel intensities of bifocal IOL (E) and trifocal IOL (F) compared to monofocal IOL (D) showed that they resulted in a greater contrast decrease.
(TIF)

S3 Fig Point light sources photographed by the artificial eye model. Red (A, D, G), blue (B, E, H), and green (C, F, I) light sources were photographed separately by using each IOL. Significant halos and glares were observed in each color case for bifocal and trifocal IOLs. (A-C) Monofocal IOL; (D-F) bifocal IOL; (G-I) trifocal IOL.
(TIF)

S4 Fig IOL PSF applied images through convolution. (TIF)

S1 File Contrast analysis and validation of holographic simulations. (PDF)

S2 File Holographic simulator assessment results and analysis. (PDF)
