# Supplementary figures and images for: Intraocular lens simulator using computational holographic display for cataract patients
Source: PLoS One. 2024 Oct 23;19(10):e0295215. doi: 10.1371/journal.pone.0295215 (PMC11498724; doi:10.1371/journal.pone.0295215)

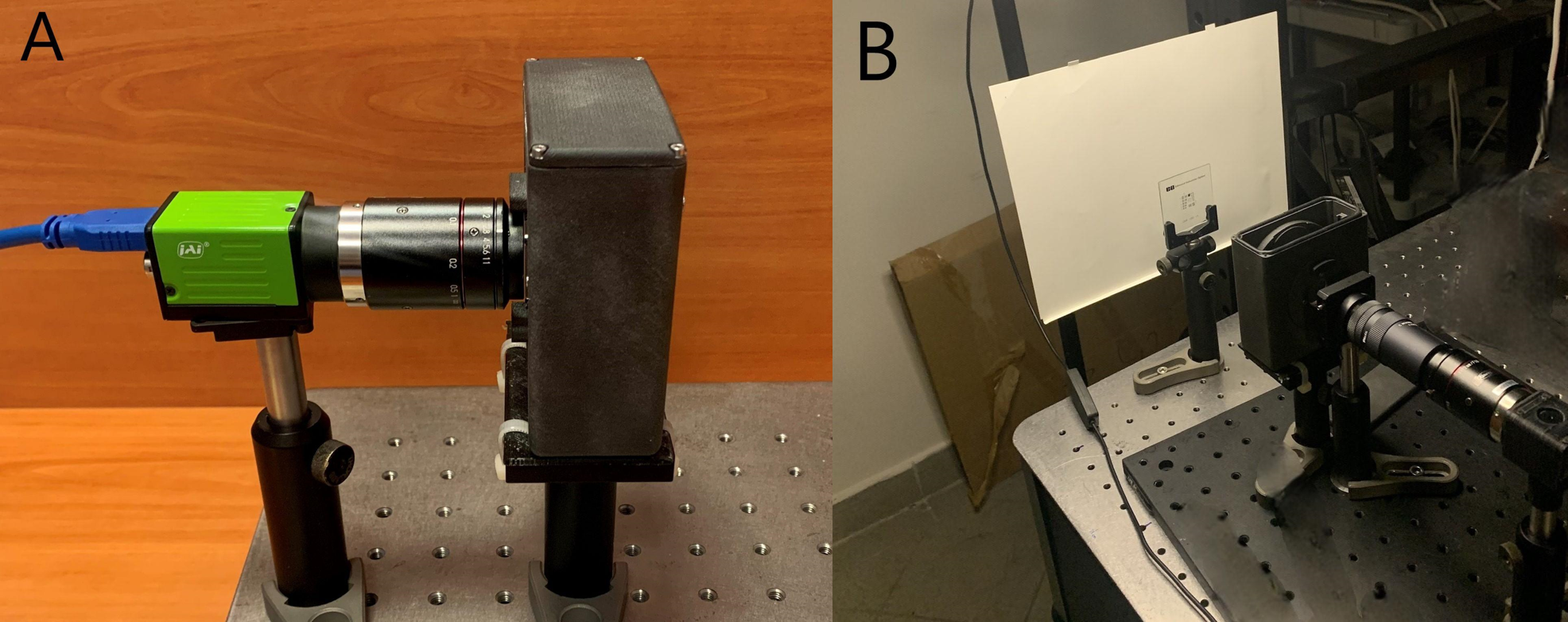

Supplement: S1 Fig — (A) A real-time capture of the scientific camera combined with an artificial eye model is given. We kept the system as compact as possible to avoid ambient light and optical aberrations from the environment. (B) An example setup used for IOL characterization. To correct the magnification, we used an artificial eye model and a camera with a x10 microscope lens. (TIF) [file pone.0295215.s001.tif]

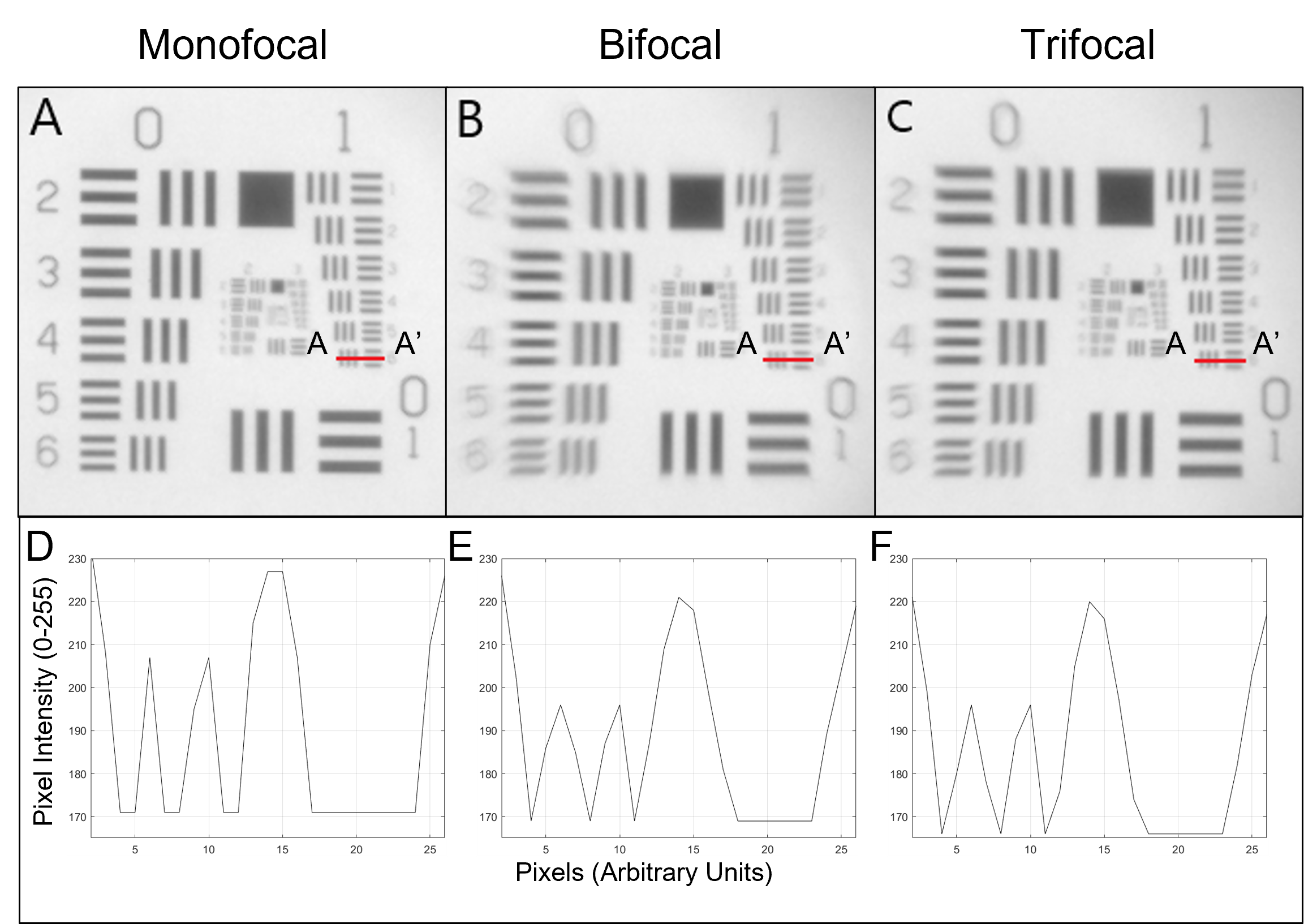

Supplement: S2 Fig — Monofocal IOL (A) had better focus compared to bifocal IOL (B) and Trifocal IOL (C). Pixel intensities of bifocal IOL (E) and trifocal IOL (F) compared to monofocal IOL (D) showed that they resulted in a greater contrast decrease. (TIF) [file pone.0295215.s002.tif]

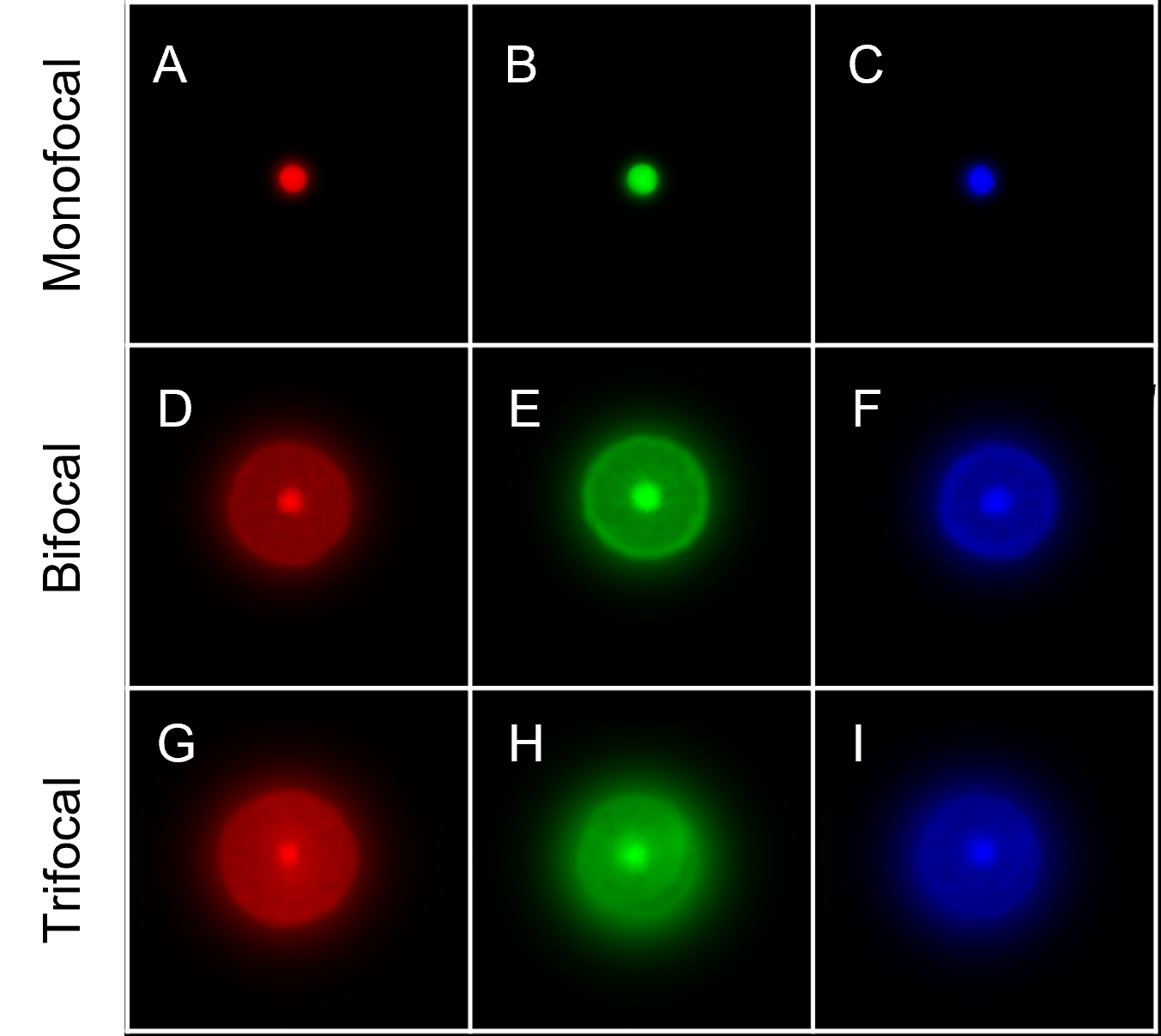

Supplement: S3 Fig — Red (A, D, G), blue (B, E, H), and green (C, F, I) light sources were photographed separately by using each IOL. Significant halos and glares were observed in each color case for bifocal and trifocal IOLs. (A-C) Monofocal IOL; (D-F) bifocal IOL; (G-I) trifocal IOL. (TIF) [file pone.0295215.s003.tif]

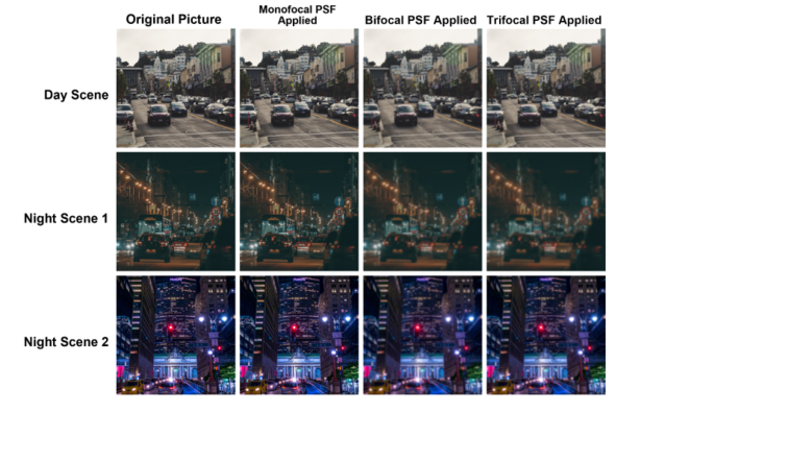

Supplement: S4 Fig — (TIF) [file pone.0295215.s004.tif]
