# Supplementary material for: Intraocular lens simulator using computational holographic display for cataract patients
Source: PLoS One. 2024 Oct 23;19(10):e0295215. doi: 10.1371/journal.pone.0295215 (PMC11498724; doi:10.1371/journal.pone.0295215)
Supplement: S1 File — (PDF) [file pone.0295215.s005.pdf]

The contrast decrease that was characterized for each IOL was applied to sine-wave gratings that correspond to their respective spatial frequencies. An example of the contrast decrease applied to 3 cpd content is shown in Figure 9. The first column represents the target content for the holography for each IOL. The second column is the real captures of holograms generated with the targets in the first column. The last column is for validation purposes, allowing us to understand if the contrast decreases match the expected results. We initially generated holograms of the same content without any contrast decrease and captured them with the artificial eye model to determine how much a physical IOL decreased the contrast of the hologram.

Figure 9.

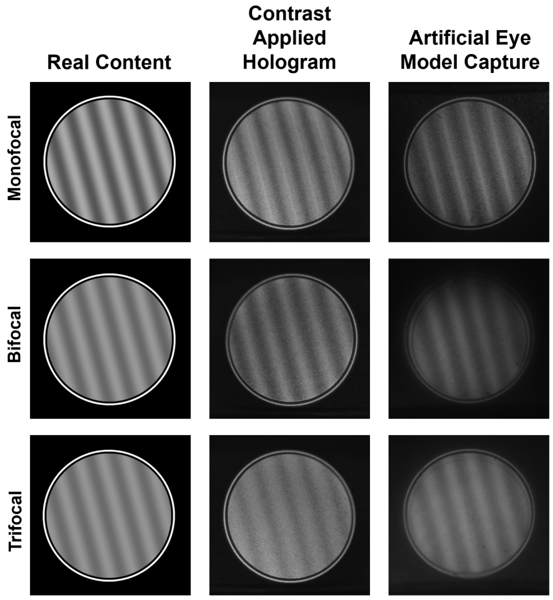

Contrast sensitivity sine-wave grating test charts with 3 cpd spatial frequency. Column1: Prepared content to mimic the contrast decrease of each IOL. Column2: Corresponding captured images in the holographic setup. Column 3: Standard test patterns (with 100% modulation) imaged through the artificial eye model using each IOL. Comparing the images in

Column 2 and Column 3, we can claim the virtual simulator using holography closely mimics the contrast reduction of the physical IOLs.

The contrast values calculated using Michelson's formula are given in Table 1. The results are very close to each other, but we see a slight underestimation in the simulated holography contrast captures compared to the captures of standard test contents through the artificial eye model. After discussing this with physicians, it was concluded that this difference was not significant for clinical applications.

Table 1.

	Prepared Content Contrast (%)	Captured Image Contrast (%)	Holography through Artificial Eye Contrast (%)
**Monofocal IOL**	32.4	32.0	33.1
**Bifocal IOL**	20.2	20.4	20.9
**Trifocal IOL**	16.0	15.8	16.0

IOL, Intraocular lens.

Contrasts in target contents and holographic results through different IOLs. Column 1 shows the contrast percentage in prepared content to mimic IOLs in holography. Column 2 shows the contrasts of the corresponding captured images in a holographic setup. In column 3, contrasts of the standard test pattern captured through the artificial eye model are given to verify simulation results.

Sine-wave grating contents were captured at various spatial frequencies to determine if the generated holograms were correctly showing contrast reduction (Fig. 10). All

holograms were captured using a scientific camera, the same as the characterization setup. The first column is the hologram captures generated with a target content corresponding to the standard test. The rest of the columns show real hologram captures that had simulated contrast reduction in their target contents, respectively for monofocal, bifocal, and trifocal IOLs.

Figure 10.

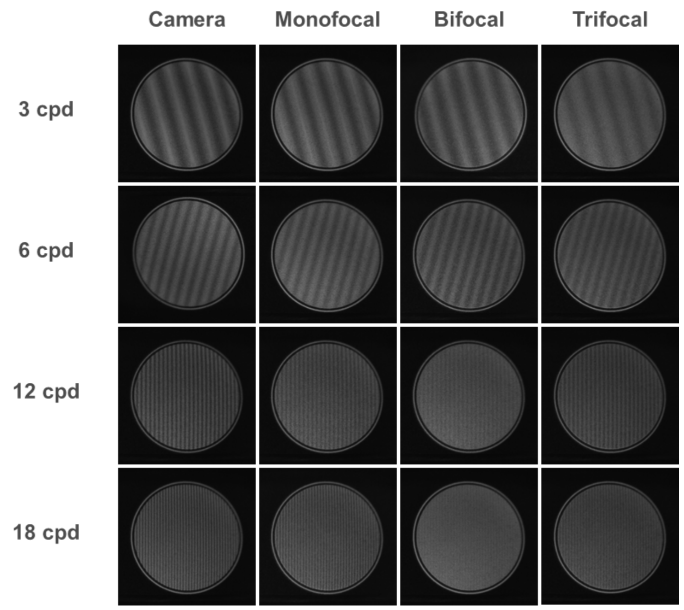

Captured images in the holographic setup using different spatial frequency fringe based holograms computed to mimic the contrast levels of different IOLs. Column 1 corresponds to using a camera lens (not an IOL). Columns 2 through 4 correspond to holograms with reduced contrast corresponding to different IOLs. Looking at the contrast reductions, we can confirm that the results match the contrast characterization made with the artificial eye model for all spatial frequencies.

Calculated contrasts of the captures in Figure 10 are given in Table 2. Monofocal IOL had a 5, 23, 47, and 52 percent contrast decrease at 3, 6, 12, and 18 cpd spatial

frequencies, respectively, compared to the expected contrast from the test. In the same contents, bifocal IOL had 38, 47, 71, and 79 percent contrast decreases, and trifocal IOLs had 35, 49, 67, and 77 percent contrast decreases. Compared to the characterization done with the artificial eye model setup (Fig. 7) for real scenes, we observe a slight but clinically insignificant underestimation in contrast decrease calculated through holography simulations.

Table 2.

	Real Contrast (%)	Monofocal Contrast (%)	Monofocal Contrast Reduction (%)	Bifocal Contrast (%)	Bifocal Contrast Reduction (%)	Trifocal Contrast (%)	Trifocal Contrast Reduction (%)
**3 cpd**	32.7	31.0	5	20.2	38	21.2	35
**6 cpd**	24.3	18.9	23	12.9	47	12.4	49
**12 cpd**	39.0	20.9	47	11.2	71	12.9	67
**18 cpd**	48.5	18.9	52	10.6	79	.5	77

Cpd, cycles per degree.

Contrasts of IOL simulated contents captured in holographic setup at different spatial frequencies based on Figure 10 and their contrast reduction relative to standard test chart holograms.
