# Supplementary material for: Intraocular lens simulator using computational holographic display for cataract patients
Source: PLoS One. 2024 Oct 23;19(10):e0295215. doi: 10.1371/journal.pone.0295215 (PMC11498724; doi:10.1371/journal.pone.0295215)
Supplement: S2 File — (PDF) [file pone.0295215.s006.pdf]

Healthy Subject Results

Figure 1A shows the defocus curves of the monofocal and multifocal IOLs using the IOL effect simulated holographic setup for healthy subjects. In the case of the monofocal IOL, the visual acuity showed a maximum at 0 D, and then it decreased continuously. In the case of the bifocal IOL, the visual acuity showed a maximum at 0 D, then slightly decreased and increased, having a second peak around -3 D. It then started decreasing again. Trifocal IOL also showed its maximum visual acuity at 0 D, compared to bifocal IOL, it had a smaller decrease and had similar peaks at -2 D and -3 D. Afterwards, visual acuity decreased. At 0 D, all IOLs showed similar performance with 0.04 (0.08), 0.04 (0.08), and 0.04 (0.06) logMAR visual acuities for monofocal, bifocal, and trifocal IOLs, respectively. At -2 D defocus, the trifocal IOL had better visual acuity, with 0.08 (0.06) logMAR compared to the other two IOLs, which had 0.26 (0.1) and 0.3 (0.02) logMAR for monofocal and bifocal IOLs, respectively. At -3 D defocus, both bifocal IOL and trifocal IOL had better visual acuity, with 0.12 (0.1) and 0.08 (0.12) logMAR, respectively, than monofocal IOL, with 0.46 (0.04) logMAR visual acuity. The trifocal IOL had slightly higher visual acuity than the bifocal IOL.

The contrast sensitivity results of sinewave grating tests with the holographic display simulator for all subjects are given in Figure 1B. As expected,in the case of the monofocal IOL, contrast sensitivity is on the higher side of the normal human range (Castellucci, 2021) , making a peak at 6 cpd spatial frequency. Contrast sensitivity curves of bifocal and trifocal IOLs follow a similar trend to monofocal IOL, both having a peak at 6 cpd spatial frequency. In comparison, although they still stay within normal human limits, they show less contrast sensitivity compared to the monofocal IOL. At lower spatial frequencies, 3 and 6 cpd, it is not possible to say any IOL has better performance than others (All p-values higher than 0.050 with a 5% confidence interval). The difference in performance between IOLs becomes significant at higher spatial frequencies. At 12 and 18 cpd spatial frequencies, monofocal IOL, with 1.99 (0.00) and 1.52 (0.00) log contrast sensitivity, had better performance than bifocal IOL, with 1.70 (1.87) and 1.18 (1.15) log contrast sensitivity ($P = 0.047$ and 0.035, Wilcoxon signed rank test), and trifocal IOL, with 1.70 (1.69) and 1.28 (1.15) log contrast sensitivity ($P = 0.020$ and 0.042, Wilcoxon signed rank test). Bifocal and trifocal IOL contrast sensitivities were not significantly comparable at any spatial frequency, showing neither of them had better performance.

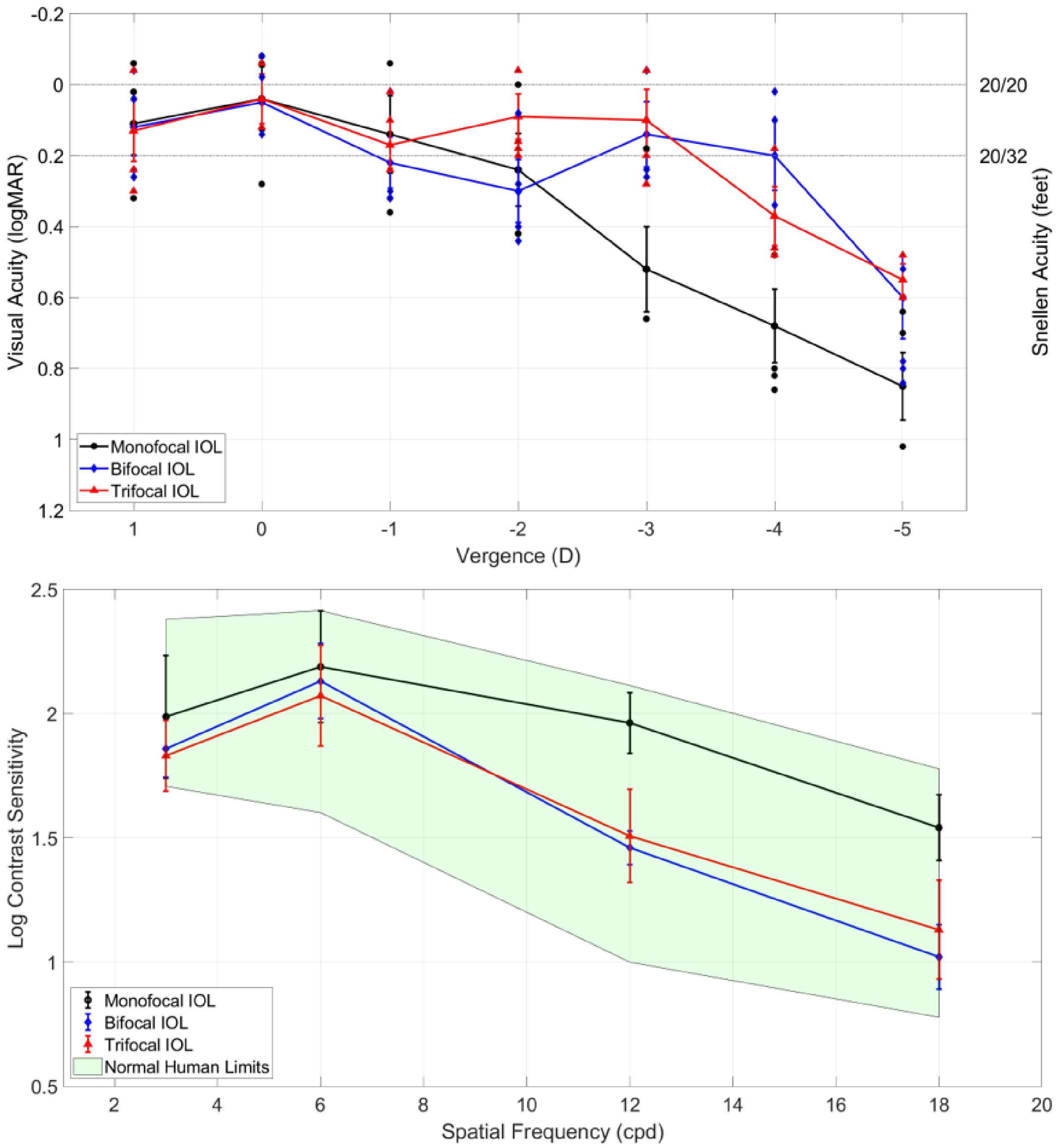

Figure 1. Defocus curves and sinewave grating contrast sensitivity curves of monofocal, bifocal, and trifocal IOLs from the visual assessments done with the holographic display simulator on healthy subjects. (A) Resulting defocus curves for different IOLs; (B) Resulting contrast sensitivity curves for healthy subject visual assessments.

Spatial Frequency (cpd)	Contrast Sensitivity (log)			p-value		
	Monofocal	Bifocal	Trifocal	Mono & Bi	Mono & Tri	Bi & Tri
3	2.08 (1.77)*	1.79 (0.00)*	1.79 (0.00)*	0.063	0.109	0.844
6	2.20 (2.11)*	1.95 (1.85)*	2.20 (1.85)*	0.109	0.320	0.2344
12	1.99 (0.00)*	1.70 (1.87)*	1.70 (1.69)*	0.047	0.020	0.203
18	1.52 (0.00)*	1.18 (1.15)*	1.28 (1.15)*	0.035	0.042	1.000

cpd: Cycles per degree
Log: Logarithm
*Median (interquartile range)

Defocus (D)	Visual Acuity (logMAR)			p-value		
	Monofocal	Bifocal	Trifocal	Mono & Bi	Mono & Tri	Bi & Tri
+1	0.1 (0.06)*	0.12 (0.08)*	0.1 (0.08)*			
0	0.04(0.08)*	0.04 (0.08)*	0.04 (0.06)*	0.594	0.762	0.344
-1	0.12 (0.02)*	0.24 (0.02)*	0.18 (0.1)*			
-2	0.26 (0.1)*	0.3 (0.02)*	0.08 (0.06)*	0.093	<0.001	<0.001
-3	0.46 (0.04)*	0.12 (0.1)*	0.08 (0.12)*	<0.001	<0.001	0.025
-4	0.56 (0.1)*	0.18 (0.14)*	0.4 (0.12)*			
-5	0.76 (0.08)*	0.6 (0.22)*	0.56 (0.08)*			

D: Diopter
LogMAR: Logarithm of the Minimum Angle of Resolution
*Median (interquartile range)

Cataract Patients' Results

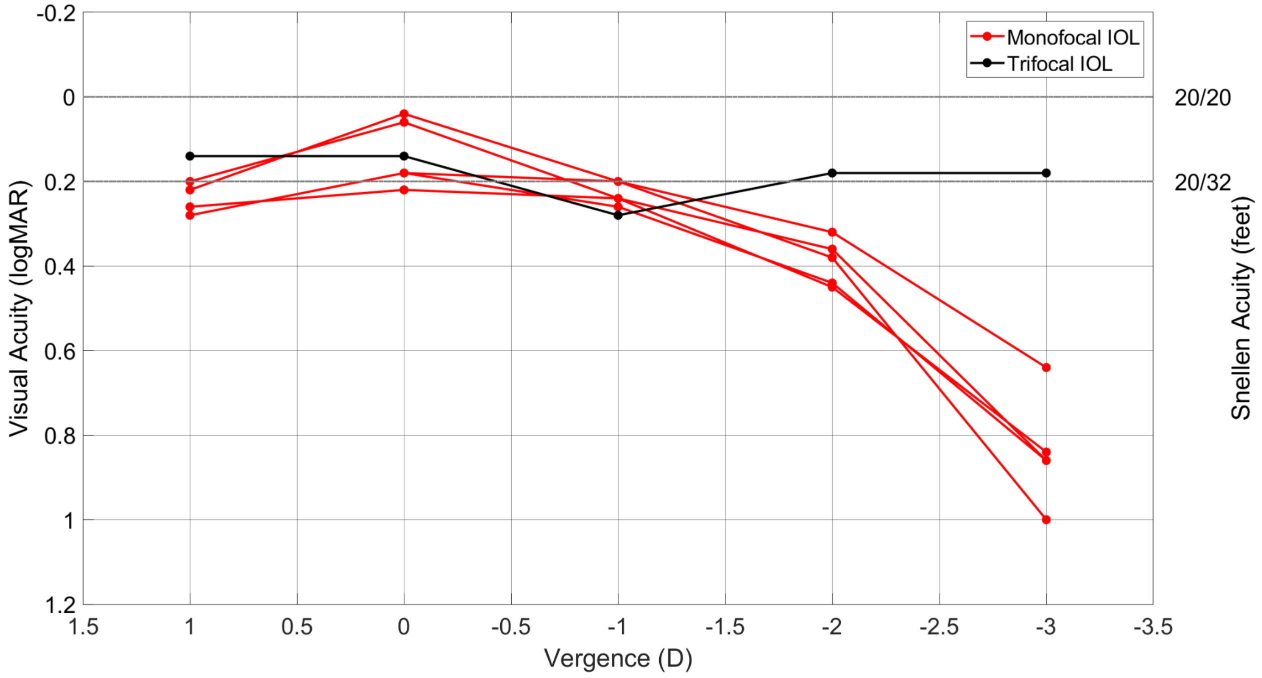

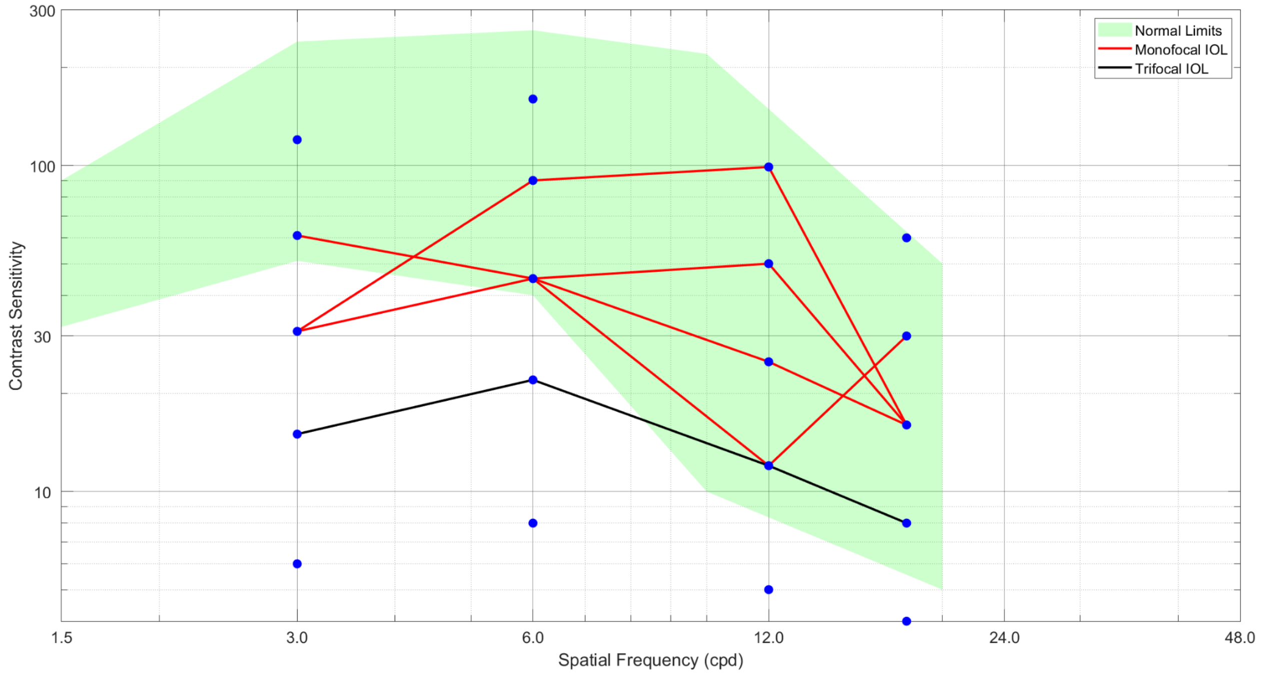

		Holographic Visual Acuity (LogMAR)				
**Patient**	**IOL Type**	**1D**	**0D**	**-1D**	**-2D**	**-3D**
**1**	**Monofocal**	0.28	0.18	0.26	0.44	0.86
**2**	**Monofocal**	0.26	0.22	0.24	0.45	0.84
**3**	**Monofocal**	0.22	0.04	0.2	0.32	0.64
**4**	**Monofocal**	0.2	0.06	0.24	0.36	0.86
**5**	**Monofocal**	0.28	0.18	0.2	0.38	1.0
**6**	**Trifocal**	0.14	0.14	0.28	0.18	0.18

		Holographic Contrast Sensitivity			
**Patient**	**IOL Type**	**3 cpd**	**6 cpd**	**12 cpd**	**18 cpd**
**1**	**Monofocal**	61	45	25	16
**2**	**Monofocal**	31	45	25	16
**3**	**Monofocal**	31	45	50	16
**4**	**Monofocal**	31	45	12	30
**5**	**Monofocal**	31	90	99	16
**6**	**Trifocal**	15	22	12	8
