# Supplementary material for: Intraocular lens simulator using computational holographic display for cataract patients
Source: PLoS One. 2024 Oct 23;19(10):e0295215. doi: 10.1371/journal.pone.0295215 (PMC11498724; doi:10.1371/journal.pone.0295215)
Supplement: Attachment — Submitted filename: Rebuttal.docx [file pone.0295215.s007.docx]

Dear Dr. Van Charles Lansingh,

We hope this message finds you well. We are writing in response to the reviewers' comments on our manuscript entitled "Intraocular lens simulator using computational holographic display for cataract patients". We appreciate the time and effort you and the reviewers have taken to evaluate our work and provide insightful feedback.

We have carefully considered all of the comments and suggestions. We believe that these revisions have significantly improved the quality and clarity of our manuscript. Below, we have responded to each comment in detail.

**1.** It is unclear for me what the main purpose of the study is. Is it the validation of the presented technology, or is it the demonstration of new technology (and if so, is it the holographic display or vision simulator), or all of it? If so, I recommend re-structuring of the manuscript, so it is easier for the reader to comprehend. Also, we should consider what is research and what is development/quality assurance/etc. To fulfil the requirements to publish the results as research, there should be a relevant research question, where the data is helping in answering the specific question. In this case, the authors state that the purpose is:” To show the expected postoperative vision to cataract patients before surgery using a vision simulator that can render the optical performance and limitations of different intraocular lens (IOL) options.” This is not a research question. A research question would for example be: “To validate a new technology for the demonstration of…”, “Does a vision simulator support the patient's decision compared to using no simulator…” (I know, this is a different study), etc. Furthermore, we have to be asking questions that are somehow generalizable, for it to be of relevance for the community. So that we can build upon previous research knowledge. As I understand, this study is a description of technology development, and you can argue that it does not fulfil the above-mentioned criteria as it is presented now. This is not the same as stating that work is not important. But in my opinion it has to be written in a way that makes it relevant for future researchers (also to reproduce and to build on the new knowledge), and with a relevant research question. Alternatively, it should be published in a journal that focuses purely on the development of new technology.

**Response:** We value your insightful comment on the main purpose of our study and the structure of our manuscript. We acknowledge that our initial manuscript may not have clearly conveyed our research question and the significance of our study. In response to your comment, we have made several revisions to our manuscript to clarify these aspects.

We have restated our purpose in lines 21-23, focusing on the development and validation of a solution for IOL simulation, rather than on technological advancement. We have introduced the problem that motivated our study in lines 50-52, highlighting the need for a technology to characterize and simulate vision through any IOL for patients prior to surgery. Our research question is now clearly stated as ‘Can holographic display-based solutions be used as a sufficient simulator for demonstrating vision through different IOLs to cataract patients’.

We have also revised our aim in lines 69-70, clarified our contributions in lines 74, 77, and 82, and extended the sentence in line 82 to explain why we conducted our testing. We have revised our discussion and conclusion sections to align with the research question we aim to answer. We have added a new sentence in lines 428-430 to clarify what our approach does and summarized our main contributions in lines 501-504. We believe our study can be generalized for multiple research purposes and have added this claim in lines 513-515.

We trust that these revisions have improved the clarity of our manuscript and made it more relevant for future researchers. We appreciate your feedback, which has helped us enhance the quality of our work.

**2.** It is unclear why those three specific IOL’s were chosen.

**Response:** We appreciate the reviewer’s query about the selection of the three specific IOLs. We acknowledge that our initial manuscript did not provide a clear rationale for this choice. In response to this comment, we have added an explanation to lines 106-108 of our revised manuscript. We have chosen these IOLs due to their wide usage in cataract surgeries, which we believe makes our findings relevant and applicable to many cases. We trust this addition clarifies our selection process.

**3.** The patient satisfaction part is unclear. Why was a scale of 1 to 5 chosen? Etc. In table 2 it is unclear whether the satisfaction data indicates the patients’ satisfaction with that specific IOL or with the result compared with the simulated vision.

**Response:** Thank you for bringing this to our attention. We apologize for any confusion caused by our initial explanation. We have made revisions in lines 309-402 to clarify this aspect. The last row of Table 2 now clearly indicates the ratings of preoperative vision satisfaction provided by cataract patients after experiencing simulated vision with the corresponding IOLs. These ratings are assessed on a scale from 1 to 5. We chose this scale because it is commonly used in ophthalmology clinics and facilitates easy collection of patient feedback. We hope this revision provides a clearer understanding of our patient satisfaction assessment.

**4.** Reg. table 1: Please state age in range (not +/- SD). Also, the table can be made as only two rows (writing the format in () e.g. age, years (mean, range).

**Response:** We appreciate the reviewer’s suggestion to improve the presentation of data in Table 1. We agree that stating the age in range and restructuring the table to only two rows would make the information more concise and clearer. We have made the suggested changes to Table 1 in our revised manuscript. Now, the age is presented in the format of ‘years (mean, range)’. We believe this revision enhances the readability of our data.

In conclusion, we would like to express our gratitude to you and the reviewers for the time and effort spent reviewing our manuscript and providing valuable feedback. We believe that these revisions have significantly improved our manuscript, making it clearer and more impactful. We look forward to your positive response.

Best regards,

Hakan Urey, PhD
