# Supplementary material for: Intraocular lens simulator using computational holographic display for cataract patients
Source: PLoS One. 2024 Oct 23;19(10):e0295215. doi: 10.1371/journal.pone.0295215 (PMC11498724; doi:10.1371/journal.pone.0295215)
Supplement: Peer review history

# Peer review: Decision Letter 0

**DOI:** 10.1371/journal.pone.0295215.r001

**Authors:** Van Charles Lansingh

8 Apr 2024

PONE-D-23-34894Intraocular lens simulator using computational holographic display for cataract patientsPLOS ONE

Dear Dr. Urey,

Thank you for submitting your manuscript to PLOS ONE. After careful consideration, we feel that it has merit but does not fully meet PLOS ONE’s publication criteria as it currently stands. Therefore, we invite you to submit a revised version of the manuscript that addresses the points raised during the review process.

Please submit your revised manuscript by May 23 2024 11:59PM. If you will need more time than this to complete your revisions, please reply to this message or contact the journal office at plosone@plos.org. When you're ready to submit your revision, log on to https://www.editorialmanager.com/pone/ and select the 'Submissions Needing Revision' folder to locate your manuscript file.

Please include the following items when submitting your revised manuscript:

- A rebuttal letter that responds to each point raised by the academic editor and reviewer(s). You should upload this letter as a separate file labeled 'Response to Reviewers'.
- A marked-up copy of your manuscript that highlights changes made to the original version. You should upload this as a separate file labeled 'Revised Manuscript with Track Changes'.
- An unmarked version of your revised paper without tracked changes. You should upload this as a separate file labeled 'Manuscript'.

If you would like to make changes to your financial disclosure, please include your updated statement in your cover letter. Guidelines for resubmitting your figure files are available below the reviewer comments at the end of this letter.

If applicable, we recommend that you deposit your laboratory protocols in protocols.io to enhance the reproducibility of your results. Protocols.io assigns your protocol its own identifier (DOI) so that it can be cited independently in the future. For instructions see: https://journals.plos.org/plosone/s/submission-guidelines#loc-laboratory-protocols. Additionally, PLOS ONE offers an option for publishing peer-reviewed Lab Protocol articles, which describe protocols hosted on protocols.io. Read more information on sharing protocols at https://plos.org/protocols?utm_medium=editorial-email&utm_source=authorletters&utm_campaign=protocols.

We look forward to receiving your revised manuscript.

Kind regards,

Van Charles Lansingh, MD, PhD

Academic Editor

PLOS ONE

Journal Requirements:

When submitting your revision, we need you to address these additional requirements.

1. Please ensure that your manuscript meets PLOS ONE's style requirements, including those for file naming. The PLOS ONE style templates can be found at 

https://journals.plos.org/plosone/s/file?id=wjVg/PLOSOne_formatting_sample_main_body.pdf and 

https://journals.plos.org/plosone/s/file?id=ba62/PLOSOne_formatting_sample_title_authors_affiliations.pdf

2. Note from Emily Chenette, Editor in Chief of PLOS ONE, and Iain Hrynaszkiewicz, Director of Open Research Solutions at PLOS: Did you know that depositing data in a repository is associated with up to a 25% citation advantage (https://doi.org/10.1371/journal.pone.0230416)? If you’ve not already done so, consider depositing your raw data in a repository to ensure your work is read, appreciated and cited by the largest possible audience. You’ll also earn an Accessible Data icon on your published paper if you deposit your data in any participating repository (https://plos.org/open-science/open-data/#accessible-data).

Additional Editor Comments:

Dear Author,

This is a very interesting and innovative paper, thank you very much. It took a long time to be able to find reviewers, hence the delay in providing you with the appropriate feedback.

Please take into consideration the comments of Reviewer 1 and re/submit. The Academic community will greatly benefit.

[Note: HTML markup is below. Please do not edit.]

Reviewers' comments:

Reviewer's Responses to Questions

**Comments to the Author**

1. Is the manuscript technically sound, and do the data support the conclusions?

The manuscript must describe a technically sound piece of scientific research with data that supports the conclusions. Experiments must have been conducted rigorously, with appropriate controls, replication, and sample sizes. The conclusions must be drawn appropriately based on the data presented. 

Reviewer #1: Partly

Reviewer #2: Yes

**********

2. Has the statistical analysis been performed appropriately and rigorously? 

Reviewer #1: Yes

Reviewer #2: Yes

**********

3. Have the authors made all data underlying the findings in their manuscript fully available?

The PLOS Data policy requires authors to make all data underlying the findings described in their manuscript fully available without restriction, with rare exception (please refer to the Data Availability Statement in the manuscript PDF file). The data should be provided as part of the manuscript or its supporting information, or deposited to a public repository. For example, in addition to summary statistics, the data points behind means, medians and variance measures should be available. If there are restrictions on publicly sharing data—e.g. participant privacy or use of data from a third party—those must be specified.

Reviewer #1: Yes

Reviewer #2: Yes

**********

4. Is the manuscript presented in an intelligible fashion and written in standard English?

PLOS ONE does not copyedit accepted manuscripts, so the language in submitted articles must be clear, correct, and unambiguous. Any typographical or grammatical errors should be corrected at revision, so please note any specific errors here.

Reviewer #1: Yes

Reviewer #2: Yes

**********

5. Review Comments to the Author

Please use the space provided to explain your answers to the questions above. You may also include additional comments for the author, including concerns about dual publication, research ethics, or publication ethics. (Please upload your review as an attachment if it exceeds 20,000 characters)

Reviewer #1: This is a well-written manuscript on an interesting topic: The use of holographic displays simulating the expected postoperative vision to cataract patients by using three different IOL’s.

However, I have some concerns regarding the methodology and presentation of the study.

Major concerns:

1. It is unclear for me what the main purpose of the study is. Is it the validation of the presented technology, or is it the demonstration of new technology (and if so, is it the holographic display or vision simulator), or all of it? If so, I recommend re-structuring of the manuscript so it is easier for the reader to comprehend.

Also, we should consider what is research and what is development/quality assurance/etc. To fulfil the requirements to publish the results as research, there should be a relevant research question, where the data is helping in answering the specific question. In this case, the authors state that the purpose is: ”To show the expected postoperative vision to cataract patients before surgery using a vision simulator that can render the optical performance and limitations of different intraocular lens (IOL) options.” This is not a research question. A research question would for example be: “ To validate a new technology for the demonstration of…”, “Does a vision simulator support the patients decision compared to using no simulator…” (I know, this is a different study), etc.

Furthermore, we have to be asking questions that are somehow generalizable, for it to be of relevance for the community. So that we can build upon previous research knowledge.

As I understand, this study is a description of technology development, and you can argue that it does not fulfil the above-mentioned criteria as it is presented now.

This is not the same as stating that the work is not important. But in my opinion it has to be written in a way that makes it relevant for future researchers (also to reproduce and to build on the new knowledge), and with a relevant research question. Alternatively, it should be published in a journal that focuses purely on the development of new technology.

2. It is unclear why those three specific IOL’s were chosen.

3. The patient satisfaction part is unclear. Why was a scale of 1 to 5 chosen? Etc. In table 2 it is unclear whether the satisfaction data indicates the patients’ satisfaction with that specific IOL or with the result compared with the simulated vision.

Minor concerns:

1. Reg. table 1: Please state age in range (not +/- SD). Also, the table can be made as only two rows (writing the format in () eg. age, years (mean, range).

Reviewer #2: I found the paper to be very interesting and well writen. I don't have any further concerns regarding ethics or dual publicación. At the moment I don't have any further recommendations for the authors.

**********

6. PLOS authors have the option to publish the peer review history of their article (what does this mean?). If published, this will include your full peer review and any attached files.

If you choose “no”, your identity will remain anonymous but your review may still be made public.

**Do you want your identity to be public for this peer review?** For information about this choice, including consent withdrawal, please see our Privacy Policy.

Reviewer #1: No

Reviewer #2: No

**********

[NOTE: If reviewer comments were submitted as an attachment file, they will be attached to this email and accessible via the submission site. Please log into your account, locate the manuscript record, and check for the action link "View Attachments". If this link does not appear, there are no attachment files.]

While revising your submission, please upload your figure files to the Preflight Analysis and Conversion Engine (PACE) digital diagnostic tool, https://pacev2.apexcovantage.com/. PACE helps ensure that figures meet PLOS requirements. To use PACE, you must first register as a user. Registration is free. Then, login and navigate to the UPLOAD tab, where you will find detailed instructions on how to use the tool. If you encounter any issues or have any questions when using PACE, please email PLOS at figures@plos.org. Please note that Supporting Information files do not need this step.

# Author comment: Author response to Decision Letter 0

**DOI:** 10.1371/journal.pone.0295215.r002

14 May 2024

Dear Dr. Van Charles Lansingh,

We hope this message finds you well. We are writing in response to the reviewers' comments on our manuscript entitled "Intraocular lens simulator using computational holographic display for cataract patients". We appreciate the time and effort you and the reviewers have taken to evaluate our work and provide insightful feedback.

We have carefully considered all of the comments and suggestions. We believe that these revisions have significantly improved the quality and clarity of our manuscript. Below, we have responded to each comment in detail.

1. It is unclear for me what the main purpose of the study is. Is it the validation of the presented technology, or is it the demonstration of new technology (and if so, is it the holographic display or vision simulator), or all of it? If so, I recommend re-structuring of the manuscript, so it is easier for the reader to comprehend. Also, we should consider what is research and what is development/quality assurance/etc. To fulfil the requirements to publish the results as research, there should be a relevant research question, where the data is helping in answering the specific question. In this case, the authors state that the purpose is:” To show the expected postoperative vision to cataract patients before surgery using a vision simulator that can render the optical performance and limitations of different intraocular lens (IOL) options.” This is not a research question. A research question would for example be: “To validate a new technology for the demonstration of…”, “Does a vision simulator support the patient's decision compared to using no simulator…” (I know, this is a different study), etc. Furthermore, we have to be asking questions that are somehow generalizable, for it to be of relevance for the community. So that we can build upon previous research knowledge. As I understand, this study is a description of technology development, and you can argue that it does not fulfil the above-mentioned criteria as it is presented now. This is not the same as stating that work is not important. But in my opinion it has to be written in a way that makes it relevant for future researchers (also to reproduce and to build on the new knowledge), and with a relevant research question. Alternatively, it should be published in a journal that focuses purely on the development of new technology.

Response: We value your insightful comment on the main purpose of our study and the structure of our manuscript. We acknowledge that our initial manuscript may not have clearly conveyed our research question and the significance of our study. In response to your comment, we have made several revisions to our manuscript to clarify these aspects.

We have restated our purpose in lines 21-23, focusing on the development and validation of a solution for IOL simulation, rather than on technological advancement. We have introduced the problem that motivated our study in lines 50-52, highlighting the need for a technology to characterize and simulate vision through any IOL for patients prior to surgery. Our research question is now clearly stated as ‘Can holographic display-based solutions be used as a sufficient simulator for demonstrating vision through different IOLs to cataract patients’.

We have also revised our aim in lines 69-70, clarified our contributions in lines 74, 77, and 82, and extended the sentence in line 82 to explain why we conducted our testing. We have revised our discussion and conclusion sections to align with the research question we aim to answer. We have added a new sentence in lines 428-430 to clarify what our approach does and summarized our main contributions in lines 501-504. We believe our study can be generalized for multiple research purposes and have added this claim in lines 513-515.

We trust that these revisions have improved the clarity of our manuscript and made it more relevant for future researchers. We appreciate your feedback, which has helped us enhance the quality of our work.

2. It is unclear why those three specific IOL’s were chosen.

Response: We appreciate the reviewer’s query about the selection of the three specific IOLs. We acknowledge that our initial manuscript did not provide a clear rationale for this choice. In response to this comment, we have added an explanation to lines 106-108 of our revised manuscript. We have chosen these IOLs due to their wide usage in cataract surgeries, which we believe makes our findings relevant and applicable to many cases. We trust this addition clarifies our selection process.

3. The patient satisfaction part is unclear. Why was a scale of 1 to 5 chosen? Etc. In table 2 it is unclear whether the satisfaction data indicates the patients’ satisfaction with that specific IOL or with the result compared with the simulated vision.

Response: Thank you for bringing this to our attention. We apologize for any confusion caused by our initial explanation. We have made revisions in lines 309-402 to clarify this aspect. The last row of Table 2 now clearly indicates the ratings of preoperative vision satisfaction provided by cataract patients after experiencing simulated vision with the corresponding IOLs. These ratings are assessed on a scale from 1 to 5. We chose this scale because it is commonly used in ophthalmology clinics and facilitates easy collection of patient feedback. We hope this revision provides a clearer understanding of our patient satisfaction assessment.

4. Reg. table 1: Please state age in range (not +/- SD). Also, the table can be made as only two rows (writing the format in () e.g. age, years (mean, range).

Response: We appreciate the reviewer’s suggestion to improve the presentation of data in Table 1. We agree that stating the age in range and restructuring the table to only two rows would make the information more concise and clearer. We have made the suggested changes to Table 1 in our revised manuscript. Now, the age is presented in the format of ‘years (mean, range)’. We believe this revision enhances the readability of our data.

In conclusion, we would like to express our gratitude to you and the reviewers for the time and effort spent reviewing our manuscript and providing valuable feedback. We believe that these revisions have significantly improved our manuscript, making it clearer and more impactful. We look forward to your positive response.

Best regards,

Hakan Urey, PhD

Attachment Submitted filename: Rebuttal.docx

# Peer review: Decision Letter 1

**DOI:** 10.1371/journal.pone.0295215.r003

**Authors:** Van Charles Lansingh

24 May 2024

Intraocular lens simulator using computational holographic display for cataract patients

PONE-D-23-34894R1

Dear Dr. Urey,

We’re pleased to inform you that your manuscript has been judged scientifically suitable for publication and will be formally accepted for publication once it meets all outstanding technical requirements.

Within one week, you’ll receive an e-mail detailing the required amendments. When these have been addressed, you’ll receive a formal acceptance letter and your manuscript will be scheduled for publication.

An invoice will be generated when your article is formally accepted. Please note, if your institution has a publishing partnership with PLOS and your article meets the relevant criteria, all or part of your publication costs will be covered. Please make sure your user information is up-to-date by logging into Editorial Manager at Editorial Manager® and clicking the ‘Update My Information' link at the top of the page. If you have any questions relating to publication charges, please contact our Author Billing department directly at authorbilling@plos.org.

If your institution or institutions have a press office, please notify them about your upcoming paper to help maximize its impact. If they’ll be preparing press materials, please inform our press team as soon as possible -- no later than 48 hours after receiving the formal acceptance. Your manuscript will remain under strict press embargo until 2 pm Eastern Time on the date of publication. For more information, please contact onepress@plos.org.

Kind regards,

Van Charles Lansingh, MD, PhD

Academic Editor

PLOS ONE

Additional Editor Comments (optional):

Reviewers' comments:

Reviewer's Responses to Questions

**Comments to the Author**

1. If the authors have adequately addressed your comments raised in a previous round of review and you feel that this manuscript is now acceptable for publication, you may indicate that here to bypass the “Comments to the Author” section, enter your conflict of interest statement in the “Confidential to Editor” section, and submit your "Accept" recommendation.

Reviewer #1: All comments have been addressed

**********

2. Is the manuscript technically sound, and do the data support the conclusions?

The manuscript must describe a technically sound piece of scientific research with data that supports the conclusions. Experiments must have been conducted rigorously, with appropriate controls, replication, and sample sizes. The conclusions must be drawn appropriately based on the data presented. 

Reviewer #1: Yes

**********

3. Has the statistical analysis been performed appropriately and rigorously? 

Reviewer #1: Yes

**********

4. Have the authors made all data underlying the findings in their manuscript fully available?

The PLOS Data policy requires authors to make all data underlying the findings described in their manuscript fully available without restriction, with rare exception (please refer to the Data Availability Statement in the manuscript PDF file). The data should be provided as part of the manuscript or its supporting information, or deposited to a public repository. For example, in addition to summary statistics, the data points behind means, medians and variance measures should be available. If there are restrictions on publicly sharing data—e.g. participant privacy or use of data from a third party—those must be specified.

Reviewer #1: Yes

**********

5. Is the manuscript presented in an intelligible fashion and written in standard English?

PLOS ONE does not copyedit accepted manuscripts, so the language in submitted articles must be clear, correct, and unambiguous. Any typographical or grammatical errors should be corrected at revision, so please note any specific errors here.

Reviewer #1: Yes

**********

6. Review Comments to the Author

Please use the space provided to explain your answers to the questions above. You may also include additional comments for the author, including concerns about dual publication, research ethics, or publication ethics. (Please upload your review as an attachment if it exceeds 20,000 characters)

Reviewer #1: (No Response)

**********

7. PLOS authors have the option to publish the peer review history of their article (what does this mean?). If published, this will include your full peer review and any attached files.

If you choose “no”, your identity will remain anonymous but your review may still be made public.

**Do you want your identity to be public for this peer review?** For information about this choice, including consent withdrawal, please see our Privacy Policy.

Reviewer #1: No

**********

# Editor report: Acceptance letter

**DOI:** 10.1371/journal.pone.0295215.r004

**Authors:** Van Charles Lansingh

15 Aug 2024

PONE-D-23-34894R1 

PLOS ONE

Dear Dr. Urey, 

I'm pleased to inform you that your manuscript has been deemed suitable for publication in PLOS ONE. Congratulations! Your manuscript is now being handed over to our production team.

At this stage, our production department will prepare your paper for publication. This includes ensuring the following:

* All references, tables, and figures are properly cited

* All relevant supporting information is included in the manuscript submission,

* There are no issues that prevent the paper from being properly typeset

If revisions are needed, the production department will contact you directly to resolve them. If no revisions are needed, you will receive an email when the publication date has been set. At this time, we do not offer pre-publication proofs to authors during production of the accepted work. Please keep in mind that we are working through a large volume of accepted articles, so please give us a few weeks to review your paper and let you know the next and final steps. 

Lastly, if your institution or institutions have a press office, please let them know about your upcoming paper now to help maximize its impact. If they'll be preparing press materials, please inform our press team within the next 48 hours. Your manuscript will remain under strict press embargo until 2 pm Eastern Time on the date of publication. For more information, please contact onepress@plos.org.

If we can help with anything else, please email us at customercare@plos.org.

Thank you for submitting your work to PLOS ONE and supporting open access. 

Kind regards, 

PLOS ONE Editorial Office Staff

on behalf of

Dr. Van Charles Lansingh 

Academic Editor

PLOS ONE
